# Endothelial Zeb2 preserves the hepatic angioarchitecture and protects against liver fibrosis

**DOI:** 10.1093/cvr/cvab148

**Published:** 2021-04-28

**Authors:** Willeke de Haan, Wouter Dheedene, Katerina Apelt, Sofiane Décombas-Deschamps, Stefan Vinckier, Stefaan Verhulst, Andrea Conidi, Thomas Deffieux, Michael W Staring, Petra Vandervoort, Ellen Caluwé, Marleen Lox, Inge Mannaerts, Tsuyoshi Takagi, Joris Jaekers, Geert Berx, Jody Haigh, Baki Topal, An Zwijsen, Yujiro Higashi, Leo A van Grunsven, Wilfred F J van IJcken, Eskeatnaf Mulugeta, Mickael Tanter, Franck P G Lebrin, Danny Huylebroeck, Aernout Luttun

**Affiliations:** Department of Cardiovascular Sciences, Center for Molecular and Vascular Biology, KU Leuven, Campus Gasthuisberg, Onderwijs & Navorsing 1, Herestraat 49, Box 911, 3000 Leuven, Belgium; Department of Cardiovascular Sciences, Center for Molecular and Vascular Biology, KU Leuven, Campus Gasthuisberg, Onderwijs & Navorsing 1, Herestraat 49, Box 911, 3000 Leuven, Belgium; Department of Internal Medicine (Nephrology), Einthoven Laboratory for Experimental Vascular Medicine, Leiden University Medical Center, Leiden, The Netherlands; Physics for Medicine Paris, Inserm, CNRS, ESPCI Paris, Paris Sciences et Lettres University, Paris, France; Department of Oncology, Laboratory of Angiogenesis and Vascular Metabolism, KU Leuven, Leuven, Belgium; Laboratory of Angiogenesis and Vascular Metabolism, Center for Cancer Biology, Vlaams Instituut voor Biotechnologie (VIB), Leuven, Belgium; Liver Cell Biology Research Group, Vrije Universiteit Brussel, Brussels, Belgium; Department of Cell Biology, Erasmus University Medical Center, Rotterdam, The Netherlands; Physics for Medicine Paris, Inserm, CNRS, ESPCI Paris, Paris Sciences et Lettres University, Paris, France; Department of Cardiovascular Sciences, Center for Molecular and Vascular Biology, KU Leuven, Campus Gasthuisberg, Onderwijs & Navorsing 1, Herestraat 49, Box 911, 3000 Leuven, Belgium; Department of Cardiovascular Sciences, Center for Molecular and Vascular Biology, KU Leuven, Campus Gasthuisberg, Onderwijs & Navorsing 1, Herestraat 49, Box 911, 3000 Leuven, Belgium; Department of Cardiovascular Sciences, Center for Molecular and Vascular Biology, KU Leuven, Campus Gasthuisberg, Onderwijs & Navorsing 1, Herestraat 49, Box 911, 3000 Leuven, Belgium; Department of Cardiovascular Sciences, Center for Molecular and Vascular Biology, KU Leuven, Campus Gasthuisberg, Onderwijs & Navorsing 1, Herestraat 49, Box 911, 3000 Leuven, Belgium; Liver Cell Biology Research Group, Vrije Universiteit Brussel, Brussels, Belgium; Department of Disease Model, Institute of Developmental Research, Aichi Developmental Disability Center, Aichi, Japan; Abdominal Surgery, UZ Leuven, Leuven, Belgium; Molecular and Cellular Oncology Laboratory, Department of Biomedical Molecular Biology, Ghent University, Ghent, Belgium; Cancer Research Institute Ghent (CRIG), Ghent, Belgium; Department of Pharmacology and Therapeutics, Rady Faculty of Health Sciences, University of Manitoba, Winnipeg, Manitoba, Canada; Research Institute in Oncology and Hematology, Cancer Care Manitoba, Winnipeg, Manitoba, Canada; Abdominal Surgery, UZ Leuven, Leuven, Belgium; Department of Cardiovascular Sciences, Center for Molecular and Vascular Biology, KU Leuven, Campus Gasthuisberg, Onderwijs & Navorsing 1, Herestraat 49, Box 911, 3000 Leuven, Belgium; Department of Disease Model, Institute of Developmental Research, Aichi Developmental Disability Center, Aichi, Japan; Liver Cell Biology Research Group, Vrije Universiteit Brussel, Brussels, Belgium; Department of Cell Biology, Erasmus University Medical Center, Rotterdam, The Netherlands; Center for Biomics-Genomics, Erasmus University Medical Center, Rotterdam, The Netherlands; Department of Cell Biology, Erasmus University Medical Center, Rotterdam, The Netherlands; Physics for Medicine Paris, Inserm, CNRS, ESPCI Paris, Paris Sciences et Lettres University, Paris, France; Department of Internal Medicine (Nephrology), Einthoven Laboratory for Experimental Vascular Medicine, Leiden University Medical Center, Leiden, The Netherlands; Physics for Medicine Paris, Inserm, CNRS, ESPCI Paris, Paris Sciences et Lettres University, Paris, France; Department of Cell Biology, Erasmus University Medical Center, Rotterdam, The Netherlands; Department of Development and Regeneration, KU Leuven, Leuven, Belgium; Department of Cardiovascular Sciences, Center for Molecular and Vascular Biology, KU Leuven, Campus Gasthuisberg, Onderwijs & Navorsing 1, Herestraat 49, Box 911, 3000 Leuven, Belgium

**Keywords:** Liver sinusoidal endothelial cells, Capillarization, Zeb2, Intussusceptive angiogenesis, Liver fibrosis

## Abstract

**Aims:**

Hepatic capillaries are lined with specialized liver sinusoidal endothelial cells (LSECs) which support macromolecule passage to hepatocytes and prevent fibrosis by keeping hepatic stellate cells (HSCs) quiescent. LSEC specialization is co-determined by transcription factors. The zinc-finger E-box-binding homeobox (Zeb)2 transcription factor is enriched in LSECs. Here, we aimed to elucidate the endothelium-specific role of Zeb2 during maintenance of the liver and in liver fibrosis.

**Methods and results:**

To study the role of Zeb2 in liver endothelium we generated EC-specific *Zeb2* knock-out (*EC^KO^*) mice. Sequencing of liver EC RNA revealed that deficiency of Zeb2 results in prominent expression changes in angiogenesis-related genes. Accordingly, the vascular area was expanded and the presence of pillars inside *EC^KO^* liver vessels indicated that this was likely due to increased intussusceptive angiogenesis. LSEC marker expression was not profoundly affected and fenestrations were preserved upon Zeb2 deficiency. However, an increase in continuous EC markers suggested that Zeb2-deficient LSECs are more prone to dedifferentiation, a process called ‘capillarization’. Changes in the endothelial expression of ligands that may be involved in HSC quiescence together with significant changes in the expression profile of HSCs showed that Zeb2 regulates LSEC–HSC communication and HSC activation. Accordingly, upon exposure to the hepatotoxin carbon tetrachloride (CCl_4_), livers of *EC^KO^* mice showed increased capillarization, HSC activation, and fibrosis compared to livers from *wild-type* littermates. The vascular maintenance and anti-fibrotic role of endothelial Zeb2 was confirmed in mice with EC-specific overexpression of Zeb2, as the latter resulted in reduced vascularity and attenuated CCl_4_-induced liver fibrosis.

**Conclusion:**

Endothelial Zeb2 preserves liver angioarchitecture and protects against liver fibrosis. Zeb2 and Zeb2-dependent genes in liver ECs may be exploited to design novel therapeutic strategies to attenuate hepatic fibrosis.

## Graphical Abstract

**Figure:**
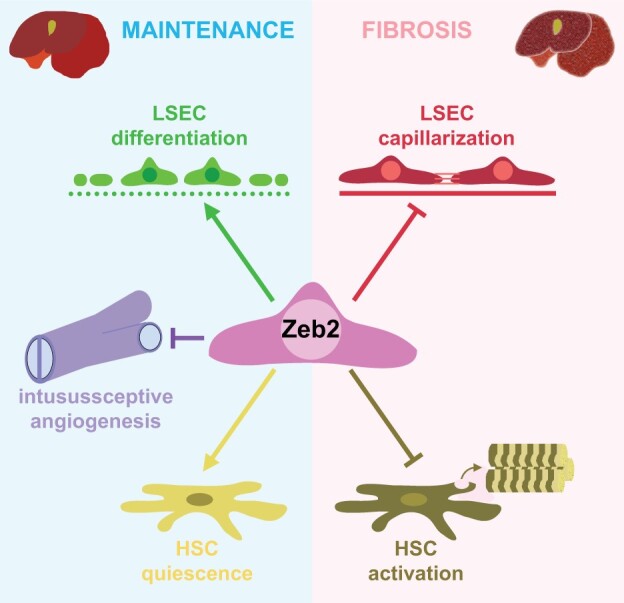

## 1. Introduction

Endothelium is highly heterogeneous between different vessel types, and capillaries are lined with specialized endothelial cells (ECs) to accommodate the specific function of the organ where they reside. Transcription factors play an important role in specialization of endothelium by enhancing the expression of proteins specific for ECs in the particular organ and/or by repressing the expression of markers for EC types found in other organs or other vessel types.^1–3^ The liver has a unique dual blood supply: a mixture of oxygen-rich hepatic arterial blood and nutrient-rich portal venous blood enters the hepatic sinusoidal capillaries. Given the importance of the liver in metabolism, detoxification, and synthesis of macromolecules, efficient transfer between blood and hepatocytes is essential. To accommodate this transfer, liver sinusoidal ECs (LSECs) are highly specialized. They have fenestrae, they lack an organized basement membrane, and they have a high receptor-mediated endocytotic capacity.^4^

Maintenance of the LSEC-specific properties is important in response to liver damage. The liver is the first organ that receives blood from the intestine and is therefore frequently exposed to noxious agents that cause liver damage. Upon liver injury, hepatic stellate cells (HSCs) become activated and deposit collagen. This leads to liver fibrosis which may ultimately progress to liver dysfunction and cirrhosis. Differentiated LSECs suppress HSC activation and thus inhibit collagen deposition and attenuate fibrosis progression.^5^ However, shortly after liver damage, LSECs start to dedifferentiate, also known as ‘capillarization’. Characteristics of capillarization are the acquisition of an organized basement membrane, loss of fenestrae, and expression of continuous EC markers such as CD31 and CD34. Upon capillarization, LSECs lose their ability to inhibit HSC activation.^3^^,^^5–7^

Several studies have established unique organ-specific EC gene signatures and showed that transcription factors enriched in liver ECs co-determine the characteristic LSEC expression profiles and functions. Our previous comparative study between ECs from heart, brain, and liver confirmed the differential expression of several known LSEC-associated transcription factors (i.e. GATA4, cMAF, and TCFEC).^2^^,^^3^^,^^8^ Furthermore, we identified new LSEC-enriched transcription factors, most notably zinc-finger E-box-binding homeobox2 (Zeb2).^8^ Zeb2 is mainly known for its role in the development of the nervous system^9–12^ and its ability to stimulate epithelial-to-mesenchymal transition (EMT) in cancer.^13^ Recent studies also suggested a role for Zeb2 in liver fibrosis by showing that Zeb2 levels are increased in fibrotic livers, and the expression of Zeb2 in HSCs has been suggested to affect their activation *in vitro.*^14^^,^^15^ Furthermore, Zeb2 maintains the tissue-specific identity of Kupffer cells and its absence leads to Kupffer cell disappearance.^16^ Since HSCs deposit collagen and Kupffer cells are involved in inflammation, these are traditionally the main cell types studied in fibrosis. More recent studies, however, emphasized the importance of LSECs, yet, the role of endothelial factors in liver fibrosis remains largely unexplored. Therefore, we aimed to elucidate the role of endothelial Zeb2 in liver vascular maintenance and its impact on fibrosis.

## 2. Methods

An extended methods section and additional references are available in Supplementary material online.

### 2.1 Mice and human tissues/cells

Four mouse lines were used: (i) EC reporter mice specifically expressing green fluorescent protein (GFP) in blood-vascular ECs (*Tie2-GFP*); (ii) *Zeb2* reporter mice expressing a Zeb2-enhanced (e)GFP fusion protein driven by the endogenous *Zeb2* promoter (*Zeb2-eGFP*); (iii) tamoxifen-inducible EC-specific *Zeb2* knock-out mice (*EC^KO^*) generated by intercrossing *Cdh5-Cre^ERT^^2^* mice with mice carrying a *Zeb2* exon 7 flanked by *lox*P sites crossed onto an R26R CAG-boosted eGFP reporter background (Supplementary material online, *Figure S1A*, left); and (iv) tamoxifen-inducible EC-specific *Zeb2*-overexpressor mice carrying the *Cdh5-Cre^ERT2^* driver and two *ROSA26-Zeb2^tg/tg^* alleles (‘*EC^OE^*’; Supplementary material online, *Figure S1A*, right). For *EC^KO^* and *EC^OE^* mice, tamoxifen-treated *Cre*-negative littermates were used as wild-type (*WT*) controls. For recombination, mice were intraperitoneally (i.p.) injected with tamoxifen for 5 consecutive days. Recombination efficiency (expressed as the fraction of ECs gaining eGFP expression) was ∼90% and similar across organs and across the zonated liver vascular bed (Supplementary material online, *Figure S1B and C*). Unless indicated otherwise, 8-week-old males were used. Blood was drawn via the heart and mice were euthanized by exsanguination under ketamine (75 μg/g i.p.) and xylazine (5 μg/g i.p.) anaesthesia. Anaesthesia depth was checked by toe pinch and, if necessary, mice received another i.p. injection with 7.5 μg/g ketamine/0.5 μg/g xylazine. Mouse experiments were approved by the KU Leuven or VUB Animal Ethics Committee and performed according to the Committee’s guidelines and those from Directive 2010/63/EU. Human liver biopsies were obtained under informed consent from patients undergoing elective cholecystectomy, fixed, paraffin-embedded, and sectioned for immunofluorescence (IF) staining. Human umbilical vein ECs (HUVECs) were isolated under informed consent from the mother. The use of human material was approved by the Ethics Committee of University Hospitals Leuven and experiments were performed according to the Committee’s guidelines and the principles of the Declaration of Helsinki.

### 2.2 Maintenance and hepatotoxic models

To study the effect of endothelial Zeb2 on (vascular) maintenance, mice were euthanized under anaesthesia as described above 1, 2, or 4 weeks after the last tamoxifen injection (Supplementary material online, *Figure S1D*). Unless indicated otherwise, mRNA expression changes are shown at 2 weeks post-tamoxifen and protein expression or structural changes at 4 weeks post-tamoxifen. To study the response to acute liver injury, mice received one i.p. injection with high-dose CCl_4_ (0.6 μL/g in mineral oil) or with mineral oil alone (vehicle) as control and were sacrificed 24 h later (Supplementary material online, *Figure S1E*). To study the effect of mild fibrosis, mice were injected with low-dose CCl_4_ (0.2 μL/g in mineral oil) or vehicle three times with 1 day in between and were sacrificed 24 h (progression cohort) or 1 week (regression or ‘R’ cohort) after the last injection (Supplementary material online, *Figure S1F*). For chronic (septal) fibrosis, mice received high-dose CCl_4_ (0.6 μL/g in mineral oil) or vehicle three times per week for 4 weeks and were sacrificed 24 h (progression cohort) or 1 week (regression or ‘R’ cohort) after the last injection (Supplementary material online, *Figure S1G*). For (immuno)histological analyses, mice were euthanized under anaesthesia as described above, livers were perfusion-fixed and processed for paraffin sectioning. For other analyses, tissues were isolated, snap-frozen, and stored until further use.

### 2.3 Cell isolation and gene profiling

To study the organ-specific *Zeb2* expression, GFP^+^ ECs were isolated from *Tie2-GFP* hearts, brains, and livers (yielding >95% pure populations consisting for >99% of microvascular ECs),^2^^,^^8^ and comparative gene expression was performed by quantitative (q) real-time (RT)-PCR. To simultaneously isolate four hepatic cell types by fluorescence-activated cell sorting (FACS) on an Aria-II sorter, *EC^KO^* and *WT* (*Cre*-negative littermates) monocellular suspensions were generated by enzymatic digestion as described.^17^ The resulting suspension was filtered and centrifuged to separate hepatocytes from the non-parenchymal cell (NPC) fraction. After lysing red blood cells, the remaining NPC fraction was stained for pan-endothelial marker Meca32 (Pan-endo) and F4/80 to enable the sorting of UV^+^ HSCs, UV^−^F4/80^+^ Kupffer cells, and UV^−^Pan-endo^+^ ECs (for antibodies: see Supplementary material online, *Table S1*). RNA was isolated using Reliaprep (Promega) and sequencing was performed by the Center for Biomics-Genomics, Erasmus MC, Rotterdam. Bioinformatic processing is described in the Supplementary material online. RNA from total liver was isolated by TRIZol. cDNA was made using the GoScript™ reverse transcription system and qRT-PCR was performed using Sybr green. *Gapdh* was used as housekeeping gene after validation (Supplementary material online, *Note S1*; for primers: see Supplementary material online, *Table S2*).

### 2.4 *In vitro* angiogenesis assays and lentivirus-mediated knock-down

Lentiviruses were made using plasmids containing *ZEB2*-shRNA or GFP in human embryonic kidney 293 cells. HUVECs were cultured in EBM2 medium supplemented with EGM2-MV in gelatin-coated flasks. Cells were transduced with viruses, and on day 6 cells were either lysed in TRIzol for RNA isolation or passaged for functional analyses (i.e. proliferation, chemotactic and scratch wound migration, tube formation, and sprouting; for details: see Supplementary material online).

### 2.5 Scanning electron microscopy and ultrafast ultrasound imaging

For morphometric analysis on vascular casts, mice were euthanized under anaesthesia and livers were perfused with VasQtec resin according to the manufacturer’s instructions. Livers were saponified and casts were dried and sputter-coated, and images were recorded on a JEOL scanning electron microscope. To document fenestrae *in vivo*, mice were perfusion-fixed with glutaraldehyde. To analyse fenestrae *ex vivo*, 36-h bulk cultures from *Zeb2 WT* and *EC^KO^* livers were fixed with glutaraldehyde. Bulk cultures were used and supplemented with 0.5 ng/mL VEGF-A in order to preserve fenestrae.^18^ Liver slices and cell cultures were dried, sputter-coated and pictures were recorded on a Zeiss Sigma VP scanning electron microscope. Ultrafast ultrasound imaging was customized for the liver, as described in Supplementary material online, and was performed on the left (lateral) liver lobe, regions of interest were selected manually based on anatomical landmarks and blood volume was computed. Spatiotemporal clutter filtering based on singular value decomposition of raw ultrasonic data were used to discriminate blood from tissue motion. Bandpass frequency filters were applied on the Doppler spectrum to give access to maps of blood flow at different velocity ranges. These ranges were related to vessel calibre based on a meta-analysis in different animal species and corresponding blood volumes in these different ranges were computed as described.^19^^,^^20^ For ultrafast ultrasound imaging, mice were anaesthetized with ketamine (75 μg/g i.p.) and medetomidine (1 μg/g once i.p.) and depth was checked by toe pinch. If necessary, mice received another i.p. injection with 7.5 μg/g ketamine and 0.1 μg/g medetomidine. After the experiment (∼45 min) anaesthesia was reversed with a subcutaneous injection of atipamezole (1 μg/g).

### 2.6 Morphometric analysis and assessment of liver fibrosis and function

For histology, mice were euthanized under anaesthesia as described above and perfusion-fixed with zinc-formalin via the heart. The left liver lobe, heart, and the brain were isolated, dehydrated, embedded in paraffin and 7 µm paraffin sections were prepared. To assess general morphology and liver damage, sections were stained with haematoxylin and eosin and collagen was revealed using Sirius red, Masson’s trichrome, or antibodies against fibrillar Collagen types I/III. To analyse the zonated architecture of the liver vasculature, we used antibodies against Cytokeratin 19 and Endomucin. To further characterize liver damage, sections were stained for α-smooth muscle actin, Desmin, and Cd45. To study endothelial changes, IF stainings were performed for Pan-endo, Cd31, Cd32, Cd34, von Willebrand factor (vWF), Laminin, Lyve1, Endomucin, *Wheat Germ Agglutinin* (WGA) lectin, and Collagen type IV. To demonstrate endothelial expression of Zeb2 in *Zeb2-eGFP* mice or to assess Cre-mediated recombination in *EC^KO^* mice, livers were co-stained for eGFP and ETS-related gene (Erg) or *Bandeiraea simplicifolia* (BS)-I lectin. To analyse recombination in periportal/pericentral LSECs, recombined ECs were counted on serial cross-sections stained for Erg/eGFP and pericentral zonation marker Cytochrome P450 Family 2 Subfamily E member 1 (Cyp2e1). Hepatocyte zonation was analysed by IF staining using antibodies against pericentral markers Cyp2e1 or glutamate ammonia ligase (Glul) and periportal/midzonal marker Arginase1 (Arg1). Where necessary, amplification was performed using Cy3- or fluorescein-tyramide kits. Human liver paraffin sections were co-stained for ZEB2 and ERG. Analyses were performed in Image J and FACS data were analysed with FACS DIVA software. Antibodies are listed in Supplementary material online, *Table S1*. To assess hydroxyproline content, livers were hydrolyzed and hydroxyproline was detected as detailed in Supplementary material online. To assess hepatocyte damage, plasma alanine transferase (ALT) was measured using a Spotchem EZ system analyser (Arkray) and GPT/ALT strips (Menarini diagnostics).

### 2.7 Statistics

Quantitative data are expressed as mean ± standard error of the mean (sem). The Student's *t*-test was used to compare two groups. ANOVA with Bonferroni *post hoc* test was used to compare more than two groups. *P-*value <0.05 was considered statistically significant. GraphPad Prism 8 was used for statistical analyses. Statistical analyses for the RNA sequencing data are described in Supplementary material online.

## 3. Results

### 3.1 Zeb2 is highly expressed in liver endothelium

**Figure 1:**
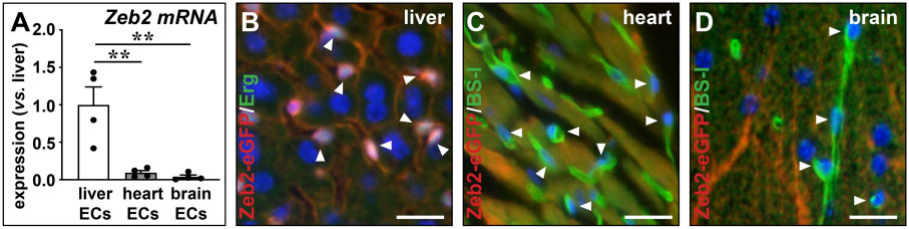
Zeb2 is highly expressed in liver endothelium. (*A*) *Zeb2* mRNA expression in ECs isolated by FACS from *Tie2-GFP* livers, hearts, and brains (*n *=* *4). (*B*–*D*) Sections of *Zeb2-eGFP* liver (*B*), heart (*C*), or brain (*D*) stained for Zeb2-eGFP (red) and EC markers Erg (green) or BS-I lectin (green). Hoechst (blue) was used as nuclear counterstain. Arrowheads indicate EC nuclei. Data are expressed as mean ± sem; **P <* *0.01 by one-way ANOVA with Bonferroni *post hoc* test. Scale bars: 50 µm. EC, endothelial cell.

In our previous microarray-based studies comparing gene expression of ECs from liver, heart, and brain, *Zeb2* emerged as a transcription factor highly enriched in liver ECs.^2^^,^^8^ To confirm this finding, we sorted ECs from liver, brain, and heart from an independent group of *Tie2-GFP* mice. GFP+ cells mainly (>99%) represent microvascular ECs.^2^^,^^8^ qRT-PCR analysis revealed high enrichment of *Zeb2* expression in liver microvascular ECs (i.e. LSECs) as compared to microvascular ECs from heart and brain (*Figure 1A*). Next, using *Zeb2-eGFP* reporter mice we showed high Zeb2-reporter activity in LSECs but not in heart or brain capillary ECs (*Figure 1B–D*). Expression of Zeb2 in LSECs was uniform across periportal and pericentral sinusoids and extended also to non-sinusoidal vessels, including the portal vein, hepatic artery, and central vein (Supplementary material online, *Figure S2A*). Finally, we stained human liver biopsies for ZEB2 and confirmed its presence in human LSECs and ECs from non-sinusoidal vessels (Supplementary material online, *Figure S2B*).

### 3.2 EC-specific *Zeb2*-KO affects expression of genes related to LSEC capillarization and LSEC–HSC communication

*Zeb2* mRNA expression was also detected in HSCs, Kupffer cells, and—to a lesser extent—in hepatocytes (Supplementary material online, *Figure S3A* and *Note S2*).^14–16^^,^^21^ To study the role of Zeb2 in the EC compartment, we generated mice, in which *Zeb2* was specifically and efficiently (∼80% at mRNA level and in ∼90% of ECs) deleted upon tamoxifen treatment in endothelium (‘*EC^KO^*’ mice; Supplementary material online, *Figures S1B and S3B and C*). Expression of *Zeb1*, the family member of *Zeb2*, was unaltered in the *EC^KO^* ECs (expression relative to *WT*: 1.0 ± 0.3 for *WT* vs. 1.0 ± 0.1 for *EC^KO^*; *n *=* *4–5). To study the consequences of EC-specific *Zeb2* deletion on gene expression in liver ECs themselves and in the neighbouring cell types in the liver sinusoids (Supplementary material online, *Figure S2A*), we isolated ECs, hepatocytes, HSCs, and Kupffer cells from *EC^KO^* and *WT* livers (Supplementary material online, *Note S3*).

**Figure 2:**
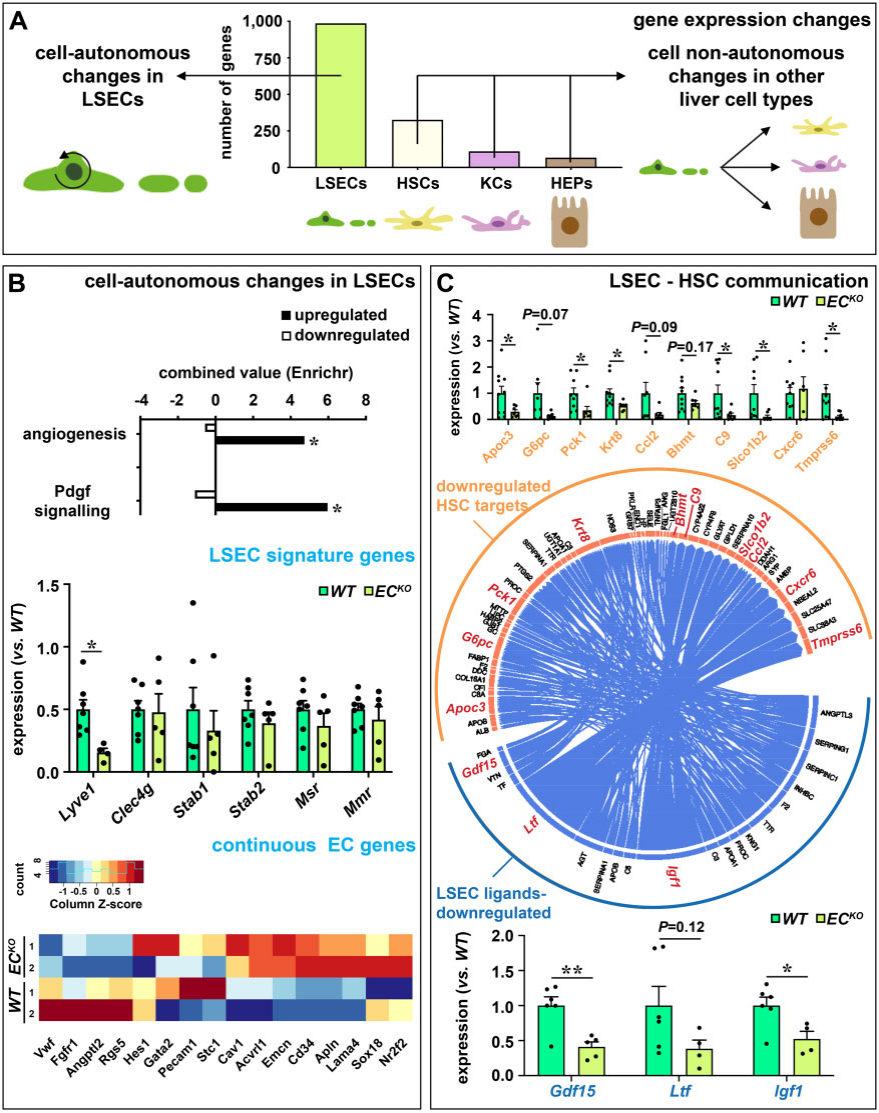
EC-specific *Zeb2*-KO alters expression of genes related to LSEC capillarization and HSC communication. (*A*) Number of differentially expressed genes in LSECs, HSCs, KCs, and HEPs (*n *=* *2). (*B*) Pathways affected by *Zeb2* (based on Enrichr analysis; *n *=* *2; top). Expression of LSEC markers (determined by qRT-PCR on an extended set of mice; *n = *5–7; middle) and continuous EC markers (heat map generated from the RNA sequencing dataset; *n = *2*;* bottom). (*C*) NicheNet-based predictions of ligands downregulated in LSEC source cells and targets downregulated in HSC target cells (*n *=* *2; middle). Expression of LSEC-derived ligand genes (bottom) and HSC target genes (top; highlighted in red in the middle diagram; determined by qRT-PCR on an extended set of mice; *n *=* *5–7). Data are expressed as mean ± sem; *P <* *0.05, **P <* *0.01 by the Student’s *t*-test. EC, endothelial cell; HEPs, hepatocytes; HSC, hepatic stellate cell; KCs, Kupffer cells; LSEC, liver sinusoidal endothelial cell; *WT*, wild-type.

Purity of the isolated cell populations was verified by qRT-PCR using acknowledged lineage markers and the different cell populations segregated according to their global expression profile determined by RNA sequencing (Supplementary material online, *Figure S4A*). The technical quality of the sequencing was verified by qRT-PCR for a random gene set on cDNA derived from RNA from the same mice (Supplementary material online, *Figure S4B*). As expected, EC-specific *Zeb2* deletion had most impact on the expression profile of LSECs themselves with 986 genes being differentially expressed (*Figure 2A* and Supplementary material online, *Figure S4C*). Functional annotation revealed that loss of *Zeb2* affected platelet-derived growth factor (Pdgf) signalling and angiogenesis (*Figure 2B*). Besides *Lyve1, Zeb2*-deletion did not profoundly affect LSEC marker expression *in vivo*, which confirmed our earlier *in vitro* observations.^8^ However, the expression of a subset of continuous EC markers^3^ including *Cd34, Endomucin* (*Emcn*), and *Apelin* (*Apln*) was increased (*Figure 2B*), indicative of early capillarization. Changes in *Lyve1* and *Emcn* were confirmed at the protein level by IF staining which revealed that their zonated expression patterns were retained upon endothelial *Zeb2* deletion. Accordingly, expression of a large number of genes with a known zonated expression pattern in LSECs was unaltered by endothelial *Zeb2* loss (Supplementary material online, *Note S4*) and preferential binding of WGA lectin in the periportal regions^22^ was also preserved (Supplementary material online, *Figure S5A–C*). Furthermore, endothelial *Zeb2* loss altered gene expression in other cell types in the liver. HSCs were most profoundly affected with differential expression of 326 genes (*Figure 2A* and Supplementary material online, *Figure S4C*). Expression changes in hepatocytes or Kupffer cells were more restricted (110 and 67 genes, respectively; *Figure 2A* and Supplementary material online, *Figure S4C*). Concomitantly, genotypes segregated for LSECs and HSCs, while they clustered randomly for hepatocytes and Kupffer cells, supporting a larger impact of the EC-specific knock-out on LSEC–HSC communication than on LSEC–hepatocyte or LSEC–Kupffer cell communication (Supplementary material online, *Figure S4A*). To further clarify the altered LSEC–HSC communication, we performed NicheNet ligand-target prediction analysis. As input criteria for this analysis we used the downregulated ligands in ECs and detectable expression of at least one corresponding receptor in the receiver cells, here HSCs. Multiple sets of downregulated target genes in HSCs were predicted that corresponded with downregulated ligands in LSECs (*Figure 2C* and Supplementary material online, *Figure S6*), supporting that LSEC–HSC communication was strongly affected by endothelial Zeb2. Downregulated LSEC ligands included growth/differentiation factor 15 (*Gdf15*),^23^ lactoferrin (*Ltf*),^24^ and insulin-like growth factor-1 (*Igf1*)^25^ which are known to attenuate liver fibrosis. We confirmed the downregulated expression of these ligands in LSECs and a subset of their fibrosis-related target genes in HSCs on an extended set of mice (*Figure 2C* and Supplementary material online, *Note S5*).

### 3.3 EC-specific *Zeb2*-KO distorts the angioarchitecture of the liver

**Figure 3:**
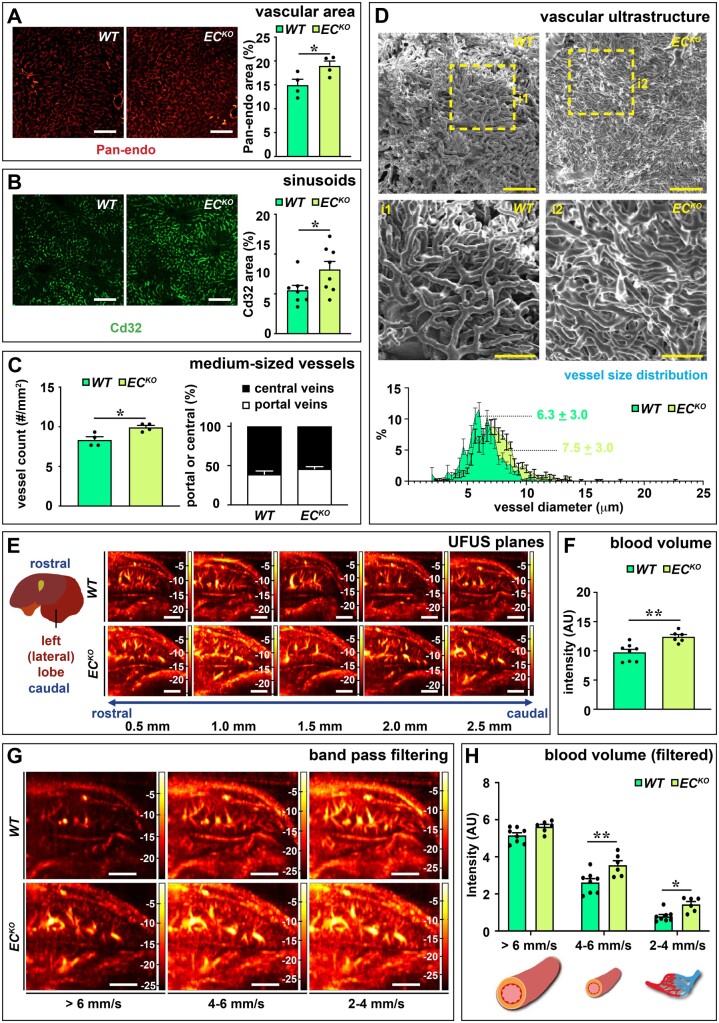
EC-specific *Zeb2*-KO distorts the angioarchitecture of the liver. (*A* and *B*) Hepatic Pan-endo^+^ (*n = *4) and Cd32^+^ area (*n = *8). (*C*) Number of medium-sized vessels counted on Pan-endo-stained sections (*left; n *=* *4) and the ratio of portal vs. central veins (*right*; *n *=* *8). (*D*) Representative scanning EM images of vascular corrosion casts and size distribution of sinusoidal diameter (*n = *7–9; *bottom*). (*E*) Representative selection of vascular planes recorded by UFUS imaging of the left (lateral) lobe. (*F*) Hepatic blood volume before bandpass filtering (*n = *6–8). (*G* and *H*) Representative Doppler images showing blood flow bandpass filtering in the region of interest for three different speed ranges, corresponding to three-vessel size ranges (*G*) and quantification of blood volume (*n = *6–8) (*H*). Data are expressed as mean ± sem, ultrafast ultrasound data are expressed in AU as mean ± sem. *P <* *0.05, **P <* *0.01 by the Student’s *t*-test (*A, B, C*, and *F*) or two-way ANOVA with Bonferroni *post hoc* test (*H*). Scale bars: 3 mm in *E* and *G*; 100 µm in *A, B*, and *D*; and 50 µm in *D*, inset. AU, arbitrary units; EC, endothelial cell; UFUS, ultrafast ultrasound; *WT,* wild-type.

Next, we investigated the consequences of endothelial loss of *Zeb2* and impaired LSEC–HSC communication on the liver phenotype 4 weeks post-tamoxifen treatment. Body and liver weight were unaffected (Supplementary material online, *Figure S7A*). Despite the perturbed LSEC–HSC communication, no signs of fibrosis were detected in unchallenged *EC^KO^* livers (Supplementary material online, *Figure S7B*). Given the altered expression profile related to angiogenesis and LSEC capillarization of *Zeb2*-deficient LSECs (*Figure 2B*), we studied the liver vasculature in detail in *EC^KO^* mice and their *WT* littermates. Pan-endo staining revealed that the total vascular surface area was significantly increased in *EC^KO^* livers at 1, 2, and 4 weeks post-tamoxifen (*Figure 3A* and Supplementary material online, *Figure S7E* and *Note S6*). The vascular expansion occurred at the level of the small Cd32^+^ sinusoidal vessels but also in the medium-sized vessels, without disturbing the ratio of portal vs. central veins (*Figure 3B and C* and Supplementary material online, *Figure S7F*). Vascular expansion was not observed in brain or heart, which was likely not due to reduced recombination efficiency but rather to the low Zeb2 levels in these tissues (Supplementary material online, *Figures S1B and S7G and H*). Since it is unknown whether the gain of continuous EC markers is associated with loss of fenestrae,^3^^,^^26^ we studied fenestration by scanning EM on liver slices and cultured liver cells and we observed that *EC^KO^* mice had no obvious defects in fenestration *in vivo* or *ex vivo*, as evidenced by a preserved porosity index (Supplementary material online, *Figure S7C and D*). These results show that LSECs only partially dedifferentiate upon *Zeb2* loss. To study how Zeb2 affects the overall ultrastructure of the vasculature, we injected mice via the portal vein with resin to create vascular corrosion casts of the liver. Analysis of these casts showed that the sinusoidal network of *EC^KO^* mice was denser and more irregular in shape with an increased vessel diameter compared to *WT* (*Figure 3D*).

To study and confirm the vascular expansion in the liver in a more integral and functional manner in RT in living mice, we customized an ultrafast ultrasound protocol for the liver. Ultrafast ultrasound allows for highly sensitive and wide-field-of-view Doppler imaging of blood vessels far beyond conventional ultrasonography (Supplementary material online, *Figure S8*).^19^ Ultrafast ultrasound on the left lateral liver lobe revealed that, while the general anatomy of the liver vascular tree remained largely preserved upon EC-specific *Zeb2-*KO (*Figure 3E*), there was an increase in blood volume in the liver of *EC^KO^* mice as compared to their *WT* littermates (*Figure 3F*). Furthermore, bandpass frequency filtering showed that the increase in blood volume was due to a significant expansion of medium-sized vessels and small (capillary/pre-capillary) vessels (with blood flow velocity of 4–6 and 2–4 mm/s, respectively;^20^  *Figure 3G and H*) which was in accordance with our histological observations.

### 3.4 Endothelial *Zeb2*-KO promotes intussusceptive angiogenesis

To learn more about the underlying mechanism of vascular expansion, we studied the direct effect of *ZEB2* knock-down (KD) *in vitro* on EC proliferation, migration, and tube formation. Since primary LSECs are hard to maintain in culture, we used HUVECs in which we silenced *ZEB2* via a lentivirus expressing a *ZEB2*-shRNA. *ZEB2*-KD did not affect proliferation, migration, or sprouting (Supplementary material online, *Figure S9A–C*). Branch formation on matrigel was, however, increased in *ZEB2*-KD HUVECs and the network was longer compared to HUVECs with unperturbed *ZEB2* expression (Supplementary material online, *Figure S9D*), suggesting the vascular expansion was due to altered EC organization rather than proliferation.

**Figure 4:**
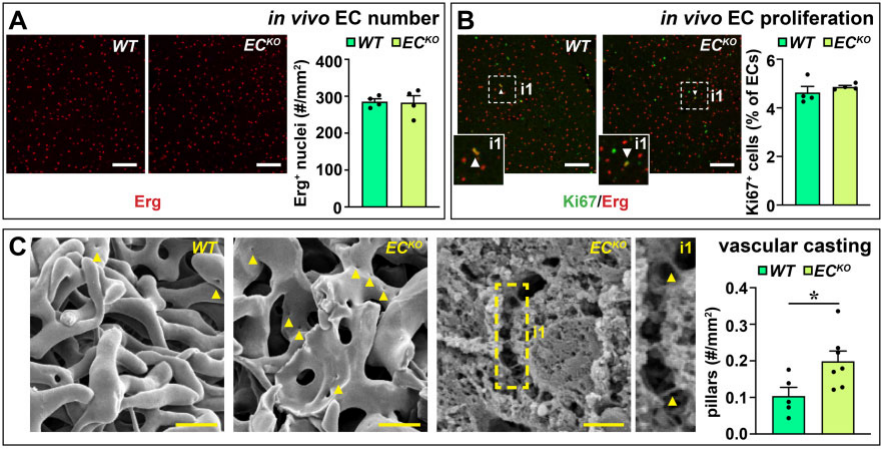
Endothelial *Zeb2*-KO promotes intussusceptive angiogenesis. (*A* and *B*) Number of Erg^+^ ECs (*A*; *n *=* *4) and % of Ki67^+^ Erg^+^ ECs (*B*; *n *=* *4). Arrowheads indicate proliferating ECs. (*C*) Representative scanning EM images of vascular corrosion casts (left) or a liver slice (middle) of an *EC^KO^* mouse and corresponding quantification (right) of number of intussusceptive pillars (*n *=* *5–7). Arrowheads indicate pillars. Data are expressed as mean ± sem; *P <* *0.05 by the Student’s *t*-test. Scale bars: 20 µm in *C, left*; 5 µm in *C*, middle. EC, endothelial cell; *WT*, wild-type.

To evaluate whether the vascular expansion *in vivo* was also independent of proliferation, we quantified the total number of ECs and proliferating ECs. EC numbers (Erg^+^ cells) were not altered in liver sections (*Figure 4A*) and accordingly, the number of Ki67^+^ Erg^+^ cells was also unaffected in livers of *EC^KO^* vs. *WT* littermates, indicating no change in proliferation (*Figure 4B*). Since non-proliferative vascular expansion is indicative of intussusceptive angiogenesis,^27^ we used vascular corrosion casts to study the presence of signs of intussusceptive angiogenesis. Interestingly, we observed a significant increase in the number of pillars, the ultrastructural hallmark of intussusceptive angiogenesis,^27^ on vascular corrosion casts of *EC^KO^* as compared to *WT* livers (*Figure 4C*), suggesting that endothelial Zeb2 modulates intussusceptive angiogenesis in the liver.

### 3.5 Endothelial *Zeb2*-KO aggravates toxin-induced liver fibrosis independent of HEP zonation

**Figure 5:**
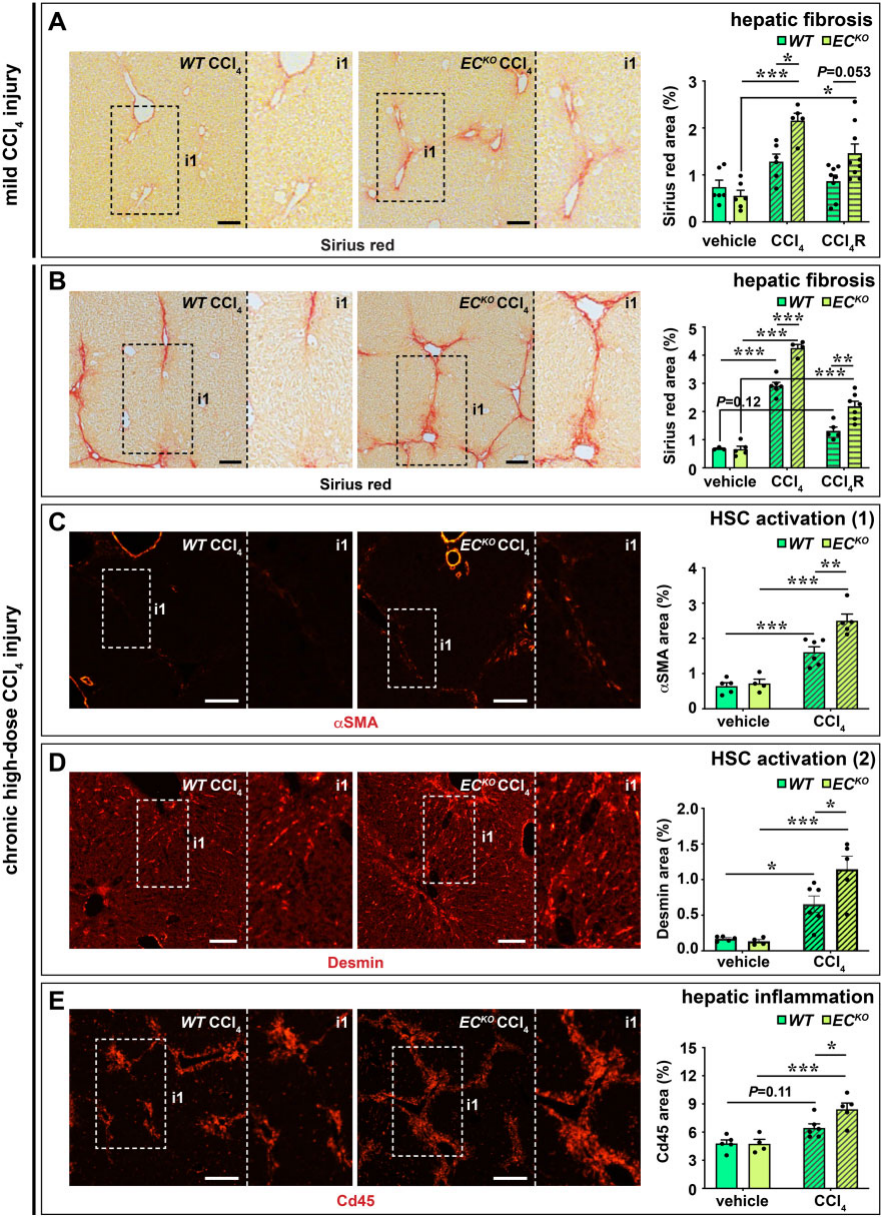
Endothelial *Zeb2*-KO promotes toxin-induced liver fibrosis. (*A*) Collagen content (Sirius red^+^ area; *n *=* *6–9) after treatment with oil (vehicle) or 1 week low-dose CCl_4_. Livers were analysed 24 h (progression cohort) or 1 week after the last oil or CCl_4_ injection (regression cohort ‘R’). (*B*) Collagen content (Sirius red^+^ area; *n *=* *3–7) after treatment with oil (vehicle) or 4 weeks high-dose CCl_4_. Livers were analysed 24 h after last oil or CCl_4_ injection (progression cohort) or 1 week after last oil or CCl_4_ injection (regression cohort ‘R’). (*C*–*E*) α-Smooth muscle actin (SMA)^+^ area (*C*; *n *=* *4–6), Desmin^+^ area (*D*; *n *=* *4–6), and Cd45^+^ area (*E*; *n *=* *4–6) after treatment with oil (vehicle) or high-dose CCl_4_. Data are expressed as mean ± sem; *P <* *0.05, **P <* *0.01, ***P <* *0.001 by one-way ANOVA with Bonferroni *post hoc* test. Scale bars: 100 µm. EC, endothelial cell; HSC, hepatic stellate cell; *WT*, wild-type.

In our RNA sequencing analysis we observed that endothelial *Zeb2* deletion changed the LSEC–HSC communication, in part by lowering the endothelial expression of ligands previously shown to attenuate fibrosis (*Figure 2B and C*). Since HSC activation is a key mediator during fibrogenesis, we hypothesized that endothelial loss of *Zeb2* would aggravate the response to a fibrotic challenge. Indeed, after a 1-week exposure to a low-dose of the hepatotoxin CCl_4_, *EC^KO^* livers had increased perivascular and parenchymal fibrosis compared to *WT* livers (*Figure 5A*). Repeated exposure to high-dose CCl_4_ for 4 weeks caused regional septal fibrosis in *WT* livers; however, septal fibrosis was more widespread in *EC^KO^* livers (*Figure 5B*). Increased fibrosis was confirmed by higher hydroxyproline content and more fibrillar Collagen type I and III deposition in CCl_4_-treated *EC^KO^* livers (Supplementary material online, *Figure S10A–C*). Accordingly, CCl_4_-treated *EC^KO^* livers had increased signs of HSC activation and inflammation in the fibrotic septa (*Figure 5C–E*). Increased fibrosis and HSC activation were also apparent from the significantly increased liver expression of genes related to these processes, including *Tgfb1, Tgfb2, Tgfb3, Col1a1, Col1a3*, and *Pdgfrb* after 4 weeks of CCl_4_ treatment (Supplementary material online, *Figure S10D*). In both mild CCl_4_ injury and chronic high-dose CCl_4_ injury, the difference in fibrosis was similar in the regression phase (i.e. 1 week after the last CCl_4_ injection; *Figure 5A and B*), indicating that endothelial Zeb2 mainly affects fibrosis progression rather than the regenerative response that occurs upon removal of the fibrotic challenge.

To study if hepatocyte damage was affected by endothelial Zeb2 we measured plasma ALT levels after a single CCl_4_ injection. ALT levels were strongly increased but similar in both genotypes indicating that the difference in fibrotic response cannot be explained by a difference in hepatocyte injury (Supplementary material online, *Figure S11A*). Since the expression of Cyp2e1—the enzyme responsible for the generation of the toxic metabolite of CCl_4_—is zonated, we investigated whether loss of endothelial *Zeb2* could affect fibrosis by skewing Cyp2e1 expression in particular and hepatocyte zonation in general. First, we found that the pattern of CCl_4_-induced necrosis was pericentral and similar in both genotypes (Supplementary material online, *Figure S11A*). Second, there was only 1 of the 67 hepatocytic DEGs (*Figure 2A* and Supplementary material online, *Figure S4C*) overlapping with a list of genes previously shown to have a zonated expression in the liver parenchyma (Supplementary material online, *Figure S11B* and *Note S7*).^28^ Third, *Zeb2* deficiency in LSECs did not alter their expression of angiocrine factors (*Wnt2, Wnt9b, Rspondin3* or *Rspo3*)^29–31^ or transcription factors (*Gata4*)^32^ known to affect hepatocyte zonation (Supplementary material online, *Figure S11B*). Finally, the zonated expression pattern of hepatocyte markers Glul, Cyp2e1, and Arg1 was preserved upon endothelial *Zeb2* loss (Supplementary material online, *Figure S11C–E*).

### 3.6 Endothelial *Zeb2*-KO aggravates toxin-induced LSEC capillarization

**Figure 6:**
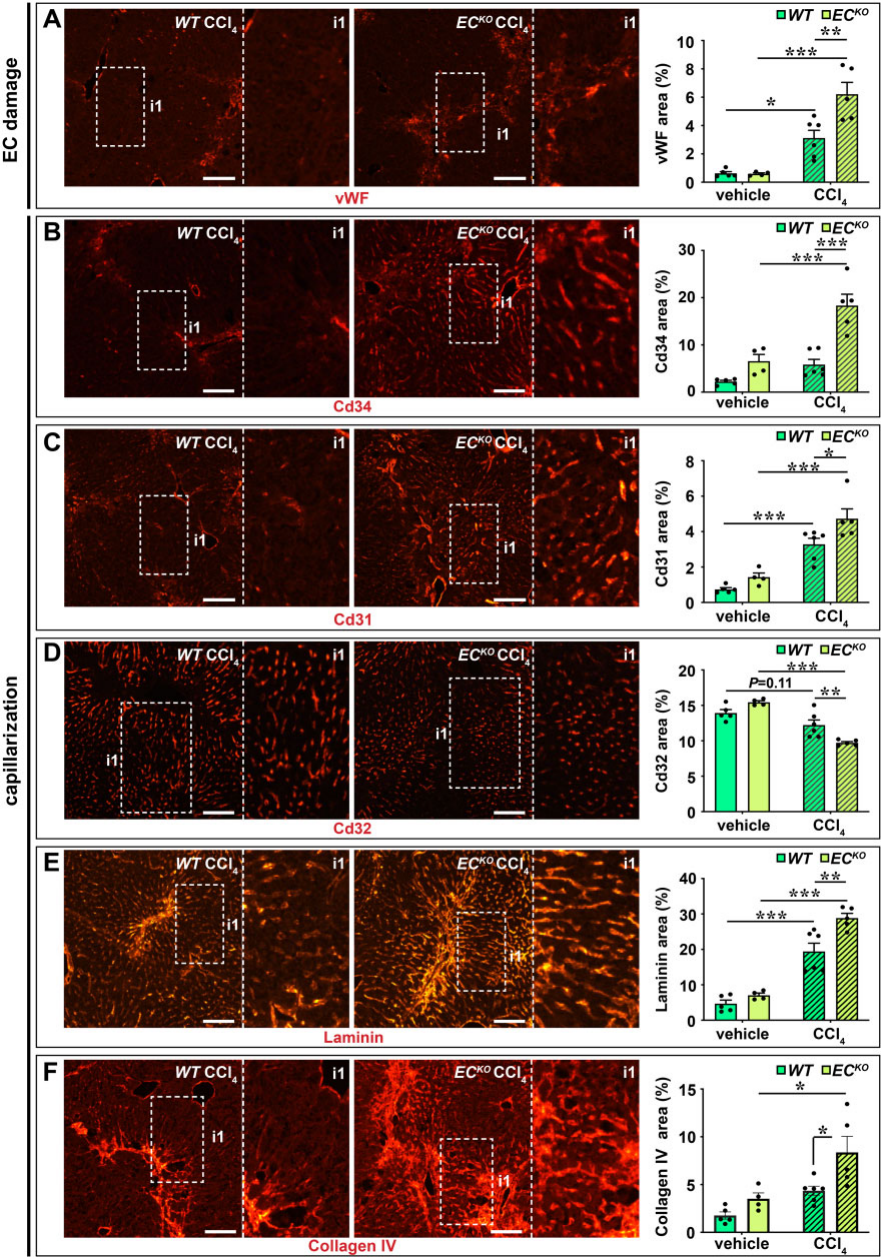
Endothelial *Zeb2*-KO promotes toxin-induced LSEC damage and capillarization. (*A*–*F*) vWF^+^ area (*A*), Cd34^+^ area (*B*), Cd31^+^ area (*C*), Cd32^+^ area (*D*), Laminin^+^ area (*E*), and Collagen type IV^+^ area (*F*) in livers after treatment with oil (vehicle) or 4 weeks high-dose CCl_4_. Data are expressed as mean ± sem; *n *=* *4–7. *P <* *0.05, **P <* *0.01, ***P <* *0.001 by one-way ANOVA with Bonferroni *post hoc* test. Scale bars: 100 µm. EC, endothelial cell; vWF, von Willebrand factor; *WT*, wild-type.

One of the early events during toxin-induced liver fibrosis is endothelial damage and capillarization which may further stimulate HSC activation and collagen deposition.^6^ In our RNA sequencing analysis we observed that endothelial *Zeb2* deletion in the non-challenged liver already increased the expression of a subset of continuous EC markers, indicative of early capillarization.^3^ Therefore, we hypothesized that capillarization of *Zeb2*-deficient LSECs would be intensified by a fibrotic challenge. Indeed, upon exposure to CCl_4_, the sinusoidal endothelium of *EC^KO^* mice showed increased vWF expression^33^ suggesting more EC damage (*Figure 6A*). Furthermore, *Zeb2 EC^KO^* livers showed more capillarization, evident from the gain of continuous EC markers Cd34 and Cd31, loss of the LSEC marker Cd32, and the acquisition of a Laminin- and Collagen Type IV-containing basement membrane (*Figure 6B–F* and Supplementary material online, *Note S8*). In agreement with the reported correlation between fibrosis progression and angiogenesis,^7^ CCl_4_-induced fibrosis was accompanied by vascular expansion (average increase in Pan-endo area of 44 ± 6%), however, this angiogenic response was not boosted by endothelial *Zeb2*-KO (Supplementary material online, *Figure S12A*). Increased capillarization was also apparent from the increased liver expression of related genes, including *Cd34, Lama1, Lama4, Col4a1*, and *Col4a2* after 4 weeks of CCl_4_ treatment (Supplementary material online, *Figure S12B*).

### 3.7 Endothelial *Zeb2-*overexpression reduces vascular expansion and toxin-induced fibrosis

**Figure 7:**
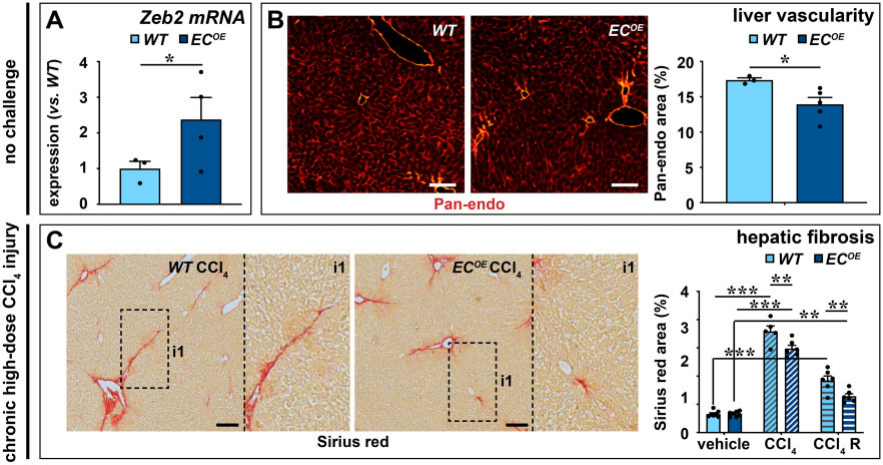
Endothelial *Zeb2-*overexpression reduces vascular expansion and toxin-induced fibrosis. (*A*) Expression of *Zeb2* in ECs from *WT* or EC-specific Zeb2 overexpressing (‘*EC^OE^*’ mice; *n *=* *5). (*B*) Pan-endo^+^ area 4 weeks after the last tamoxifen injection (*n *=* *3–5). (*C*) Collagen content (Sirius red^+^ area; *n *=* *5–7) mice 24 h after the last injection of oil or CCl_4_ (progression cohort) or 1 week after the last CCl_4_ injection (regression cohort ‘R’). Data are expressed as mean ± sem; *P <* *0.05, **P <* *0.01, ***P <* *0.001 by the Student’s *t*-test (*A* and *B*) or one-way ANOVA with Bonferroni *post hoc* test (*C*). Scale bars: 100 µm. EC, endothelial cell; *WT*, wild-type.

Our data suggest that endothelial Zeb2 preserves normal liver angioarchitecture and protects against fibrosis. To directly demonstrate this via a complementary approach, we generated mice that conditionally (Cre-dependently) overexpress *Zeb2* in ECs (‘*EC^OE^*’ mice; *Figure 7A* and Supplementary material online, *Figure S1A*, right). Like endothelial *Zeb2-*KO, gain of endothelial *Zeb2* expression did not affect body or liver weight (Supplementary material online, *Figure S13A*). *Zeb2*-overexpression in ECs caused reduced vascularity in unchallenged mice (*Figure 7B* and Supplementary material online, *Figure S13B* and *Note S9*), and fibrosis was attenuated in *EC^OE^* mice mildly or chronically challenged with CCl_4_ (*Figure 7C* and Supplementary material online, *Figure S13C*) further confirming our observations in *EC^KO^* mice and directly supporting the anti-fibrotic role of Zeb2 in LSECs.

## 4. Discussion

Our organs are equipped with a microvascular network specifically adapted to support the organ's function. Identifying what determines these specific endothelial features and how they change during pathological conditions is important to design procedures to diagnose and treat organ-specific diseases. Here, we show that high levels of the transcription factor Zeb2 are expressed in the liver endothelium as compared to endothelium in heart and brain. Endothelial Zeb2 preserved the hepatic angioarchitecture and attenuated the fibrogenic response upon (chronic) exposure to a hepatotoxin.

A key observation of our study was that *Zeb2* deletion in ECs of adult mice caused the liver vasculature to expand. Even though we used a generalized approach to delete *Zeb2* in ECs, vascular expansion did not occur in heart or brain, which is in agreement with the very low levels of Zeb2 in endothelium in these organs and an organ-specific role for Zeb2 in the liver. In a previous study using a *Tie2-Cre* driver line to conditionally delete *Zeb2* in ECs and haematopoietic cells, no major defects of the developing liver vasculature were found but mice died *in utero* because of brain haemorrhage due to the *Zeb2* deletion in the haematopoietic lineage.^34^ Here, we used a more EC-specific and tamoxifen-inducible Cre driver line (*Cdh5-Cre^ERT^^2^*) to be able to study the role in liver ECs in adult mice. Our detailed microscopic and ultrastructural analyses of *EC^KO^* mice did reveal expansion of the adult liver's vasculature and significant irregularities in the angioarchitecture.

Previously, it was suggested that *ZEB2* has a pro-angiogenic effect; however, the effects of *ZEB* were indirectly evaluated^35^ or only observed upon high-glucose exposure *in vitro*.^36^ We found that KD of *ZEB2* in HUVECs did not affect their proliferative, migratory, or sprouting behaviour but did increase their ability to branch. Complementary to our *in vitro* findings, endothelial *Zeb2*-KO did not affect EC proliferation *in vivo* in the liver, but the presence of pillars suggested the induction of a non-sprouting/non-proliferative type of angiogenesis, known as intussusceptive angiogenesis.^27^ Intussusceptive angiogenesis can cause rapid vascular expansion,^27^ which is compatible with our observation that EC-*Zeb2*-deficient mice showed vascular expansion within 1 week after deletion. The molecular mechanisms underlying intussusceptive angiogenesis remain largely unknown, although a number of candidate regulators have been identified including Pdgf-b^37^ and Notch1.^38^  *Pdgfb* was one of the genes upregulated by *Zeb2-*KO in ECs while in Schwann cells Zeb2 is needed to generate anti-Notch activity.^11^^,^^12^ While the aberrant liver angioarchitecture of *EC^KO^* mice shared some features (e.g. large variation in sinusoidal diameter) with that in *Notch1*-deficient livers, expression of *Notch1* or Notch target genes *Hey1* and *Hes1* were not significantly altered in *Zeb2*-deficient ECs (Supplementary material online, *Figure S14A*), suggesting that they work independently in liver ECs. Therefore, *Zeb2*-*EC^KO^* mice could be an interesting mouse model to further elucidate mechanisms of intussusceptive angiogenesis.

To obtain a more global and functional view on the liver vasculature, we optimized an ultrafast ultrasound protocol previously applied in brain^19^ for the use in liver, which is a soft tissue subject to significant movement, both intrinsic and extrinsic, the latter due to breathing. For this purpose, we applied a complimentary two-dimensional motion correction algorithm. Then, by applying bandpass filtering on the Doppler spectrum in each individual pixel, we showed an increase in blood volume mainly in the smaller/medium-sized vessels of *EC^KO^* livers, in accordance with our microscopic/ultrastructural findings.

Another key observation of our study was that endothelial Zeb2 protects against fibrosis in the liver. As such, our study further underscores that Zeb2 has a tissue, cell type- and context-dependent role in fibrosis. Zeb2 contributes to fibrosis in the heart by stimulating cardiac fibroblast-myofibroblast phenoconversion.^39^ Zeb2 expression has been shown to increase upon CCl_4_-exposure which may be related to non-EC cell types in the liver, including HSCs. In HSCs, Zeb2 has been suggested to have a pro-fibrotic role since its downregulation through mir145 overexpression caused apoptosis and inhibited activation of the HSC LX-2 cell line.^14^^,^^15^ Zeb2 also has a complex role in hepatocytes where *Zeb2* deficiency supports fat accumulation but also enhances liver regeneration via increased hepatocyte proliferation.^14^^,^^15^^,^^21^ In Kupffer cells, Zeb2 has been shown to be an important determinant of their specific identity and differentiation state.^16^ Zeb2 did not affect the zonated expression pattern of LSECs but rather caused a partial LSEC dedifferentiation under baseline conditions. This dedifferentiation (known as ‘capillarization’) was more pronounced upon CCl_4_ challenge. This aggravated capillarization likely contributed to the increased fibrosis upon endothelial *Zeb2* loss, as capillarized LSECs lose their ability to inhibit HSC activation.^6^ The different and sometimes opposite roles for Zeb2 in different liver cell types definitely implies that using Zeb2 as a target for treating liver disease will require a cell type-specific approach. Here, we studied the role of Zeb2 upon toxin-induced liver fibrosis. It remains to be determined whether endothelial Zeb2 would have a similar role in other liver injury models.

Zeb2 has extensively been studied in cancer because of its role in primary tumours and metastasis by promoting EMT.^13^^,^^16^ ECs can also change to a mesenchymal cell phenotype through a similar process, known as endothelial-to-mesenchymal transition (EndoMT).^40^ EndoMT has been suggested to play a role in liver fibrosis since *Erg*-deletion in mice was recently shown to lead to EndoMT and liver fibrosis; the extent to which EndoMT occurs during fibrosis is, however, organ-dependent and is limited in the liver.^40–42^ If Zeb2 would induce EndoMT, its loss in ECs would reduce fibrosis. Based on mRNA expression changes in *Zeb2*-deficient ECs, we found no evidence for a role of Zeb2 in EndoMT (Supplementary material online, *Figure S14B*).

Depending on which co-factors Zeb2 interacts with, it can either act as transcriptional repressor or activator.^9^ Zeb2 acts mainly as a transcriptional repressor in embryonic stem cells, haematopoietic cells, forebrain neurons, and myelinating cells,^11^^,^^12^^,^^43–46^ but loss of *Zeb2* in ECs revealed a majority of downregulated genes (Supplementary material online, *Figure S4C*). This could be because of its direct aforementioned action as a transcriptional activator through interaction with activating co-factors and/or indirectly occur via Zeb2-dependent repression of EC-specific other repressors, a mechanism that has also been suggested in Schwann cells.^11^^,^^12^ While the pro-angiogenic and dedifferentiation effects resulting from endothelial *Zeb2-*KO were likely mostly the result of a cell-autonomous effect, we propose that the pro-fibrotic effect was related to the disturbed paracrine communication with HSCs. A non-autonomous effect has been observed previously in brain cortex development, where Zeb2 present in the upper layers of neurons is needed for the control of appropriate levels of factors that control neurogenesis and gliogenesis in subventricular progenitor cells^45^ and deletion of Zeb2 from the ventricular-subventricular zone in the developing brain affects non-targeted cells.^10^ In contrast to HSCs, endothelial *Zeb2*-KO had the least effect on the expression profiles of hepatocytes and did not change the expression of angiocrine factors known to be involved in hepatocyte zonation,^29–31^ suggesting the communication with hepatocytes was not much affected. Also, endothelial *Zeb2* loss did not influence the hepatocytes’ regenerative response during the regression phase nor did it alter the intrinsic susceptibility of hepatocytes to CCl_4_-induced acute damage. Accordingly, endothelial *Zeb2* deficiency did not disturb the zonated expression pattern of hepatocyte markers, including that of Cyp2e1, the enzyme responsible for the metabolization of CCl_4_ that is required for its hepatotoxicity. In contrast, a recent study in which Gata4, another LSEC-enriched transcription factor, was deleted in LSECs revealed altered paracrine communication with both hepatocytes and HSCs, the former involving decreased angiocrine signals supporting hepatocyte regeneration and zonation and the latter involving increased expression of *Pdgfb*, which is known to activate HSCs.^32^ We also found increased *Pdgfb* expression upon endothelial *Zeb2* deletion, which may co-determine the increased fibrosis seen in *Zeb2-EC^KO^* mice. In addition, our NicheNet analysis predicted numerous downregulated ligands in LSECs that have previously been shown to attenuate liver fibrosis (including *Gdf15, Igf1*, and *Ltf*), hence their concerted downregulation could stimulate HSC activation upon *Zeb2-*KO in ECs.^23–25^ Although the functional importance of these ligands is supported by our observation that a large panel of their predicted targets was also downregulated in HSCs, additional experiments are required to prove their causal involvement in altered HSC behaviour upon endothelial *Zeb2* deletion. While endothelial *Gata4* deficiency caused spontaneous fibrosis, we did not observe this upon endothelial *Zeb2* loss, even after long-term follow-up (Supplementary material online, *Note S10*), suggesting that compared to *Gata4* deficiency, *Zeb2* loss had a less severe phenotype. This aligns with our previous report^8^ that Gata4 has more impact on the LSEC signature than Zeb2; however, the difference could also partly be due to a different timing of the deletion which was during the embryonic stage in the Gata4 study^32^ vs. the adult stage in ours.

Endothelial *Zeb2-*KO did not provoke spontaneous fibrosis, but aggravated fibrosis upon repeated toxin exposure. We therefore propose that while endothelial *Zeb2*-KO can sensitize HSCs by releasing the brake on their activation, an additional challenge is necessary to induce full HSC activation and fibrosis. While endothelial *Zeb2*-deficiency only induced partial signs of spontaneous LSEC capillarization, upon exposure to CCl_4_, the loss of *Zeb2* strongly aggravated this process. Finally, in line with the previous observation that fibrosis is associated with vascular expansion,^7^ exposure to CCl_4_ caused an expansion of the vasculature; however, this expansion was not further increased by endothelial *Zeb2*-KO. Therefore, the pro-fibrotic effect of endothelial *Zeb2*-KO is not likely indirectly caused by vascular expansion and the effect of *Zeb2*-KO on vascular expansion was only apparent in the non-challenged liver. Altogether, we therefore conclude that the increased fibrosis in *EC^KO^* mice was most likely due to increased capillarization and altered EC-HSC communication resulting in increased HSC activation.

In conclusion, we demonstrate that Zeb2 has a cell type-specific and context-dependent role in liver vascular maintenance and fibrosis. Our study also emphasizes the importance of the liver endothelium in the pathogenesis of liver fibrosis. Currently, there are no effective treatments for patients suffering from liver fibrosis, hence the identification of novel candidate targets is urgent. To exploit the protective effect of Zeb2 in ECs against liver fibrosis and bypass its pro-fibrogenic effect in other liver cells, an EC-specific approach should be designed. Alternatively, the HSC quiescence factors we identified that depend on Zeb2 in ECs could be used as new means to tackle fibrosis.

## Supplementary material

Supplementary material is available at *Cardiovascular Research* online.

## Authors’ contributions

W.d.H. designed the study, performed experiments, analysed data, and wrote the manuscript; W.D. performed experiments, analysed data, and edited the manuscript; K.A. performed ultrafast ultrasound experiments and analysed data; A.C., M.W.S., S.Vi., P.V., E.C., M.L., and I.M. performed experiments. W.F.J.v.IJ. coordinated RNA sequencing experiments. S.D.-D., T.D., and M.T. optimized ultrafast ultrasound data processing and analysis; S.Ve. performed NicheNet analysis; E.M. performed RNA sequencing data analysis; T.T. and Y.H. provided a transgenic mouse model and performed experiments; J.J. and B.T. provided human biopsies; G.B. and J.H. provided transgenic mouse models; A.Z., L.A.v.G., and F.P.G.L. provided critical intellectual input; D.H. provided transgenic mouse models and critical intellectual input; A.L. designed the study and edited the manuscript. All authors reviewed and approved the manuscript.

**Conflict of interest:** none declared.

## Funding

This work was supported by internal funding from KU Leuven [C12/16/023 to W.d.H. and A.Z., C14/19/095 to A.L., A.Z., and D.H., and ID-N19/031 to A.L.]; a Program Financing grant [PF/10/014 to A.L.]; a European Research Council grant [FP7-StG-IMAGINED203291 to A.L.]; a Cosmetics Europe/European Commission FP7 Grant [FP7-Health-HemiBio266777 to A.L. and L.A.v.G.]; an Interuniversity Attraction Poles grant [IUAP/P7/07 to A.L., A.Z., and D.H.]; Fonds voor Wetenschappelijk Onderzoek [FWO] grants [G.0A3116 to D.H. and W001420N to A.L., F.P.G.L., and A.Z.]; a ZONMW OffRoad program [2018/23115|ZONMW to E.M.]; a grant of KAKENHI [17K09896 to Y.H.]; a Marie Sklodowska-Curie Actions post-doctoral fellowship [H2020-MSCA-IF-REZONABLE658666 to W.d.H.], a Horizon 2020 Marie Skłodowska-Curie Actions-Innovative Training Network grant [H2020-MSCA-ITN-RenalToolBox813839 to F.P.G.L. and K.A.]; a pre-doctoral FWO fellowship [1157318N to W.D.]; a post-doctoral FWO fellowship [12N5419N LV 2479 to I.M.]; the Inserm Technology Research Accelerator grant in Biomedical Ultrasound [to M.T.]; and a grant from the Dutch Cancer Society [KWF 10339 to F.P.G.L.].

## Data availability

The RNA sequencing datasets underlying this article are available in the NCBI GEO repository at https://www.ncbi.nlm.nih.gov/geo/, and can be accessed with GSE150699.

## Supplementary Material

cvab148_Supplementary_Data

## Notes

Translational perspective Liver sinusoidal endothelial cells (LSECs) are specialized to support transport between blood and hepatocytes and to protect against fibrosis by inhibiting hepatic stellate cell (HSC) activation. Here, we show that transcription factor Zeb2 in LSECs controls their specialization and communication with HSCs and protects against fibrosis. Therefore, Zeb2 and Zeb2-dependent genes represent appealing targets to tackle liver fibrosis.
