# Supplementary material for: Endothelial Zeb2 preserves the hepatic angioarchitecture and protects against liver fibrosis
Source: Cardiovasc Res. 2021 Apr 28;118(5):1262–75. doi: 10.1093/cvr/cvab148 (PMC8953454; doi:10.1093/cvr/cvab148)
Supplement: cvab148_Supplementary_Data [file cvab148_supplementary_data.pdf]

CVR-2020-1567R2

SUPPLEMENTARY INFORMATION

Endothelial Zeb2 preserves the hepatic angioarchitecture and protects against liver fibrosis

Willeke de Haan[1], Wouter Dheedene[1], Katerina Apelt[2], Sofiane Décombas-Deschamps[3], Stefan Vinckier[4,5], Stefaan Verhulst[6], Andrea Conidi[7], Thomas Deffieux[3], Michael W. Staring[1], Petra Vandervoort[1], Ellen Caluwé[1], Marleen Lox[1], Inge Mannaerts[6], Tsuyoshi Takagi[8], Joris Jaekers[9], Geert Berx[10,11], Jody Haigh[12,13], Baki Topal[9], An Zwijsen[1], Yujiro Higashi[8], Leo A. van Grunsven[6], Wilfred F.J. van IJcken[7,14], Eskeatnaf Mulugeta[7], Mickael Tanter[3], Franck P.G. Lebrin[2,3], Danny Huylebroeck[7,15] and Aernout Luttun[1]

[1]Center for Molecular and Vascular Biology, Department of Cardiovascular Sciences, KU Leuven, Leuven, Belgium; [2]Department of Internal Medicine (Nephrology), Einthoven Laboratory for Experimental Vascular Medicine. Leiden University Medical Center. Leiden, The Netherlands; [3]Physics for Medicine Paris, Inserm, CNRS, ESPCI Paris, Paris Sciences et Lettres University, Paris, France; [4]Department of Oncology, Laboratory of Angiogenesis and Vascular Metabolism, KU Leuven, Leuven, Belgium; [5]Laboratory of Angiogenesis and Vascular Metabolism, Center for Cancer Biology, Vlaams Instituut voor Biotechnologie (VIB), Leuven, Belgium; [6]Liver Cell Biology research group, Vrije Universiteit Brussel, Brussels, Belgium; [7]Department of Cell Biology, Erasmus University Medical Center, Rotterdam, The Netherlands; [8]Department of Disease Model, Institute of Developmental Research, Aichi Developmental Disability Center, Aichi, Japan; [9]Abdominal Surgery, UZ Leuven, Leuven, Belgium; [10]Molecular and Cellular Oncology Laboratory, Department of Biomedical Molecular Biology, Ghent University, Ghent, Belgium; [11]Cancer Research Institute Ghent (CRIG), Ghent, Belgium; [12]Department of Pharmacology and Therapeutics, Rady Faculty of Health Sciences, University of Manitoba, Winnipeg, Manitoba, Canada; [13]Research Institute in Oncology and Hematology, Cancer Care Manitoba, Winnipeg, Manitoba, Canada; [14]Center for Biomics-Genomics, Erasmus University Medical Center, Rotterdam, The Netherlands; [15]Department of Development and Regeneration, KU Leuven, Leuven, Belgium

A. SUPPLEMENTARY FIGURES

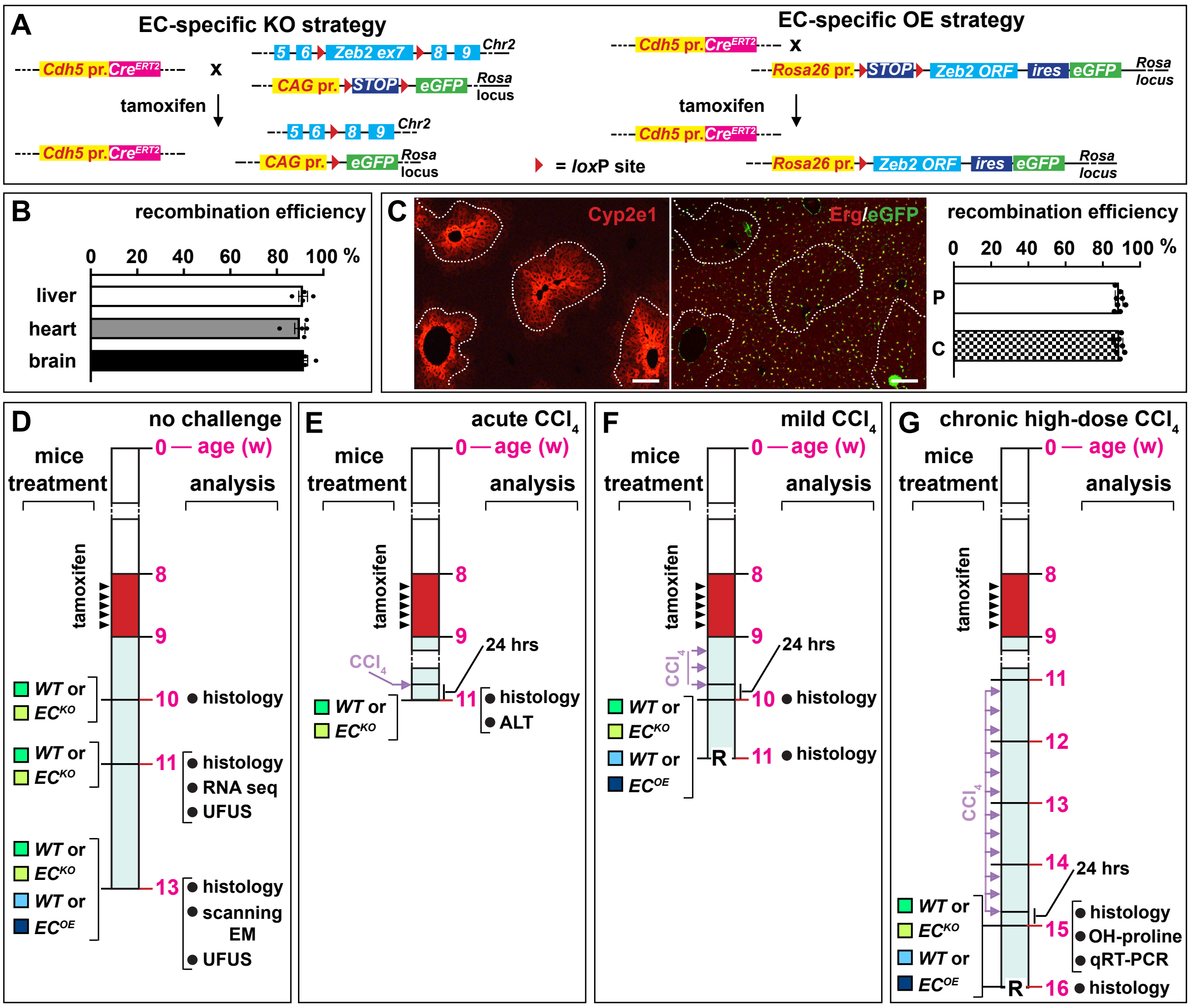

Figure S1. Transgenic mouse models for EC-specific *Zeb2* expression manipulation and study design. (A) Schematic diagram showing the genetic strategy for EC-specific *Zeb2*-knock-out (KO; *A,left*) or EC-specific *Zeb2*-overexpression (OE; *A,right*). eGFP: enhanced green fluorescent protein; pr.: promoter; CAG: CMV early enhancer/chicken beta actin; ires: internal ribosome entry site; ORF: open reading frame. **(B)** Recombination efficiency (expressed as fraction of recombined eGFP$^+$ cells of the total number of endothelial cells) of the *Cdh5-CreERT2* driver in liver (*n*=4), heart (*n*=5) and brain (*n*=5). **(C)** Representative images of serial cross-sections of livers stained with the pericentral hepatocyte marker Cyp2e1 (*left*) and a combination (*right*) of endothelial marker Erg (red)/recombination marker eGFP (green) and corresponding quantification of recombination efficiency in the Cyp2e1$^+$ pericentral ('C'; lined by white dashed lines) *vs.* Cyp2e1$^-$ periportal ('P') zone (*n*=8). Data are expressed as mean ± sem. **(D-G)** Schematic diagrams showing the study design for evaluating the role of Zeb2 during no challenge ('maintenance') (*D*), acute carbon-tetrachloride (CCl_4) challenge (*E*), mild CCl_4 exposure (*F*) or chronic CCl_4 exposure (*G*). Types of analyses performed are shown on the *right*, the timing is displayed in the *middle* and the mouse strains and treatment regimens used are mentioned on the *left*. w: weeks; hrs: hours; ALT: alanine transferase; qRT-PCR: quantitative real-time polymerase chain reaction; EC^{KO}: endothelial-specific *Zeb2*-knock-out; EC^{OE}: endothelial-specific *Zeb2*-overexpression; OH-proline: hydroxyproline; 'R': regression analysis time point; EM: electron microscopy; UFUS: ultrafast ultrasound. Scale bars: 100 µm.

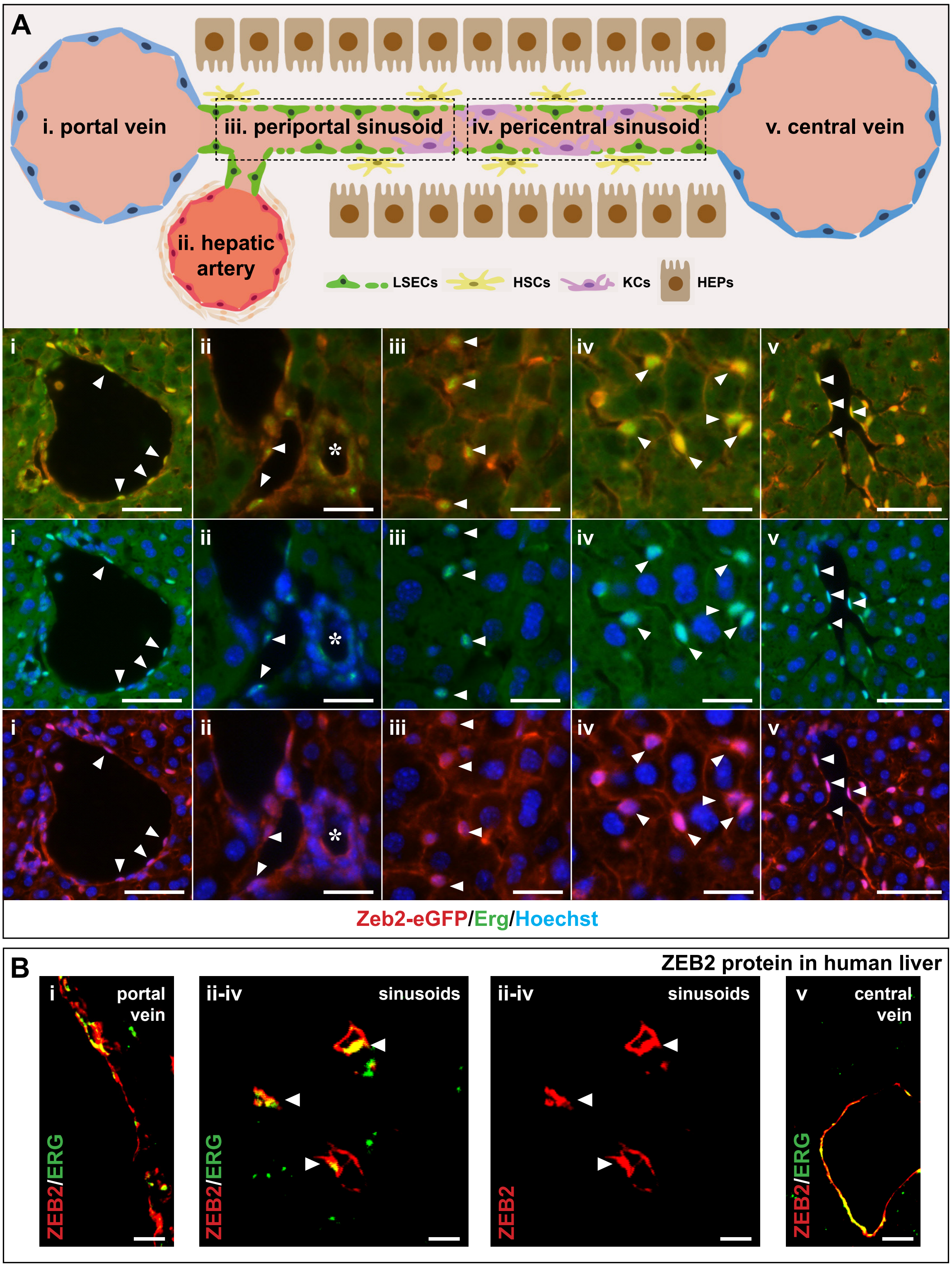

Figure S2. Zeb2 is widely expressed in liver endothelium in mice and humans. (A) Schematic representation of a liver sinusoid (*top*) and sections (*bottom*) of each region (*i-v*) of *Zeb2-eGFP* livers stained for Zeb2-eGFP (red), EC marker Erg (green) or a combination of Zeb2-eGFP (red) an EC marker Erg (green). Nuclei are stained with Hoechst (blue). Arrowheads indicate Zeb2-expressing ECs. Asterisks indicate bile duct. **(B)** Confocal images of human liver sections of the portal triad (*i*), sinusoidal region (*ii-iv*) and central vein (*v*) stained for a nuclear EC marker (ERG; green) and ZEB2 (red). Arrowheads indicate co-localisation of ZEB2 and ERG signals. Scale bars: 50 µm in *Ai,v*; 20 µm in *Aii-iv*; 10 µm in *Bi+iii;* and 5 µm in *Bii*.

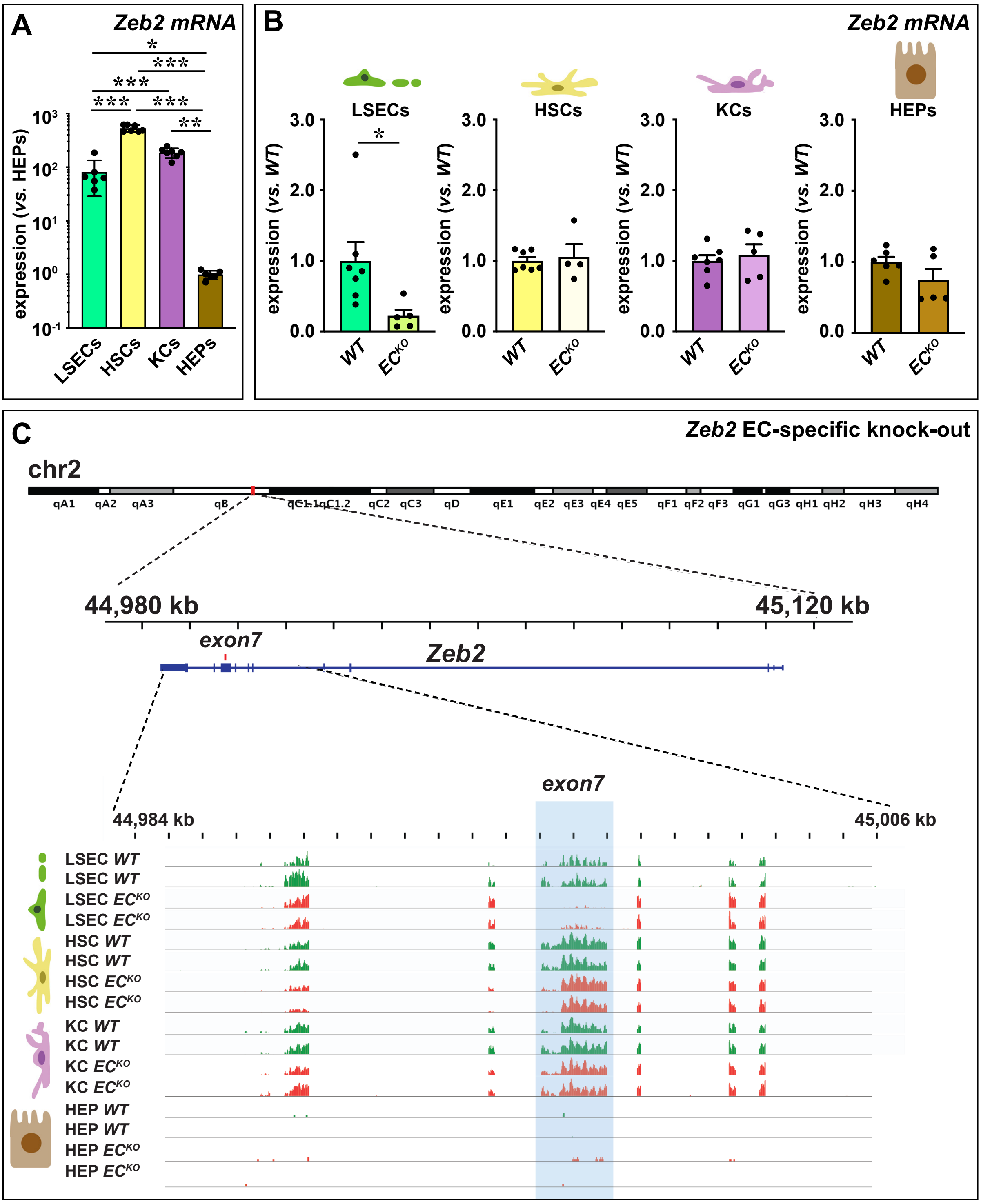

Figure S3. EC-specific *Zeb2* knock-out. (A) *Zeb2* mRNA expression in LSECs, HSCs, Kupffer cells (KCs) and hepatocytes (HEPs) of *WT* livers (n=5-7). **(B)** *Zeb2* mRNA expression in LSECs, HSCs, KCs and HEPs from *WT* and EC^{KO} mice (n=5-7). **(C)** Schematic representation of mouse chromosome 2, red mark indicates the *Zeb2* locus. The *bottom* diagram zooms in on the region around *exon 7* (which is the part of the gene that is deleted upon tamoxifen-induced Cre-mediated recombination). The RNA sequencing coverage is shown for each of the samples included in the study set-up, revealing that *exon7* reads are exclusively diminished in LSECs in the EC^{KO} mice. Note that the expression of *Zeb2* detected by RNA sequencing in HEPs is very low. Data represent mean ± sem; **P*<0.05, ***P*<0.01, ****P*<0.001.

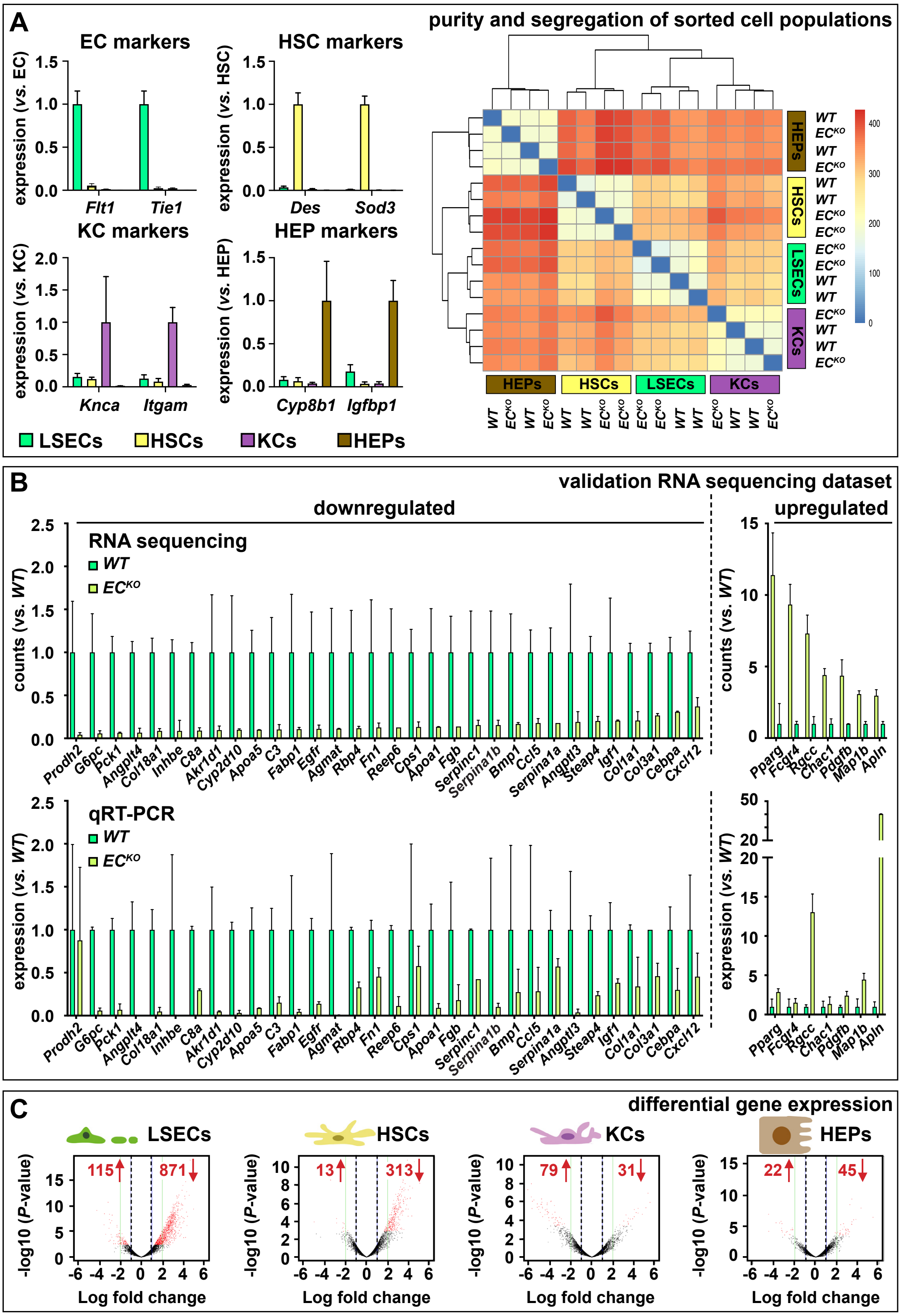

Figure S4. Purity and segregation of sorted cell populations, validation of RNA sequencing data and differential gene expression. (A) Expression (n=5-6) of cell-type-specific markers for ECs, HSCs, Kupffer cells (KCs), and hepatocytes (HEPs) (*left*) and cluster heat map (*right*) of samples included in the RNA sequencing analysis. **(B)** Expression derived from the RNA sequencing expressed as mean counts relative to *WT* and corresponding expression determined by quantitative (q)RT-PCR (n=2). **(C)** Volcano plots showing genes up- or downregulated in LSECs, HSCs, KCs and HEPs upon EC-specific *Zeb2*-knock-out. Data represent mean ± sem (*A*) or mean ± SD (*B*).

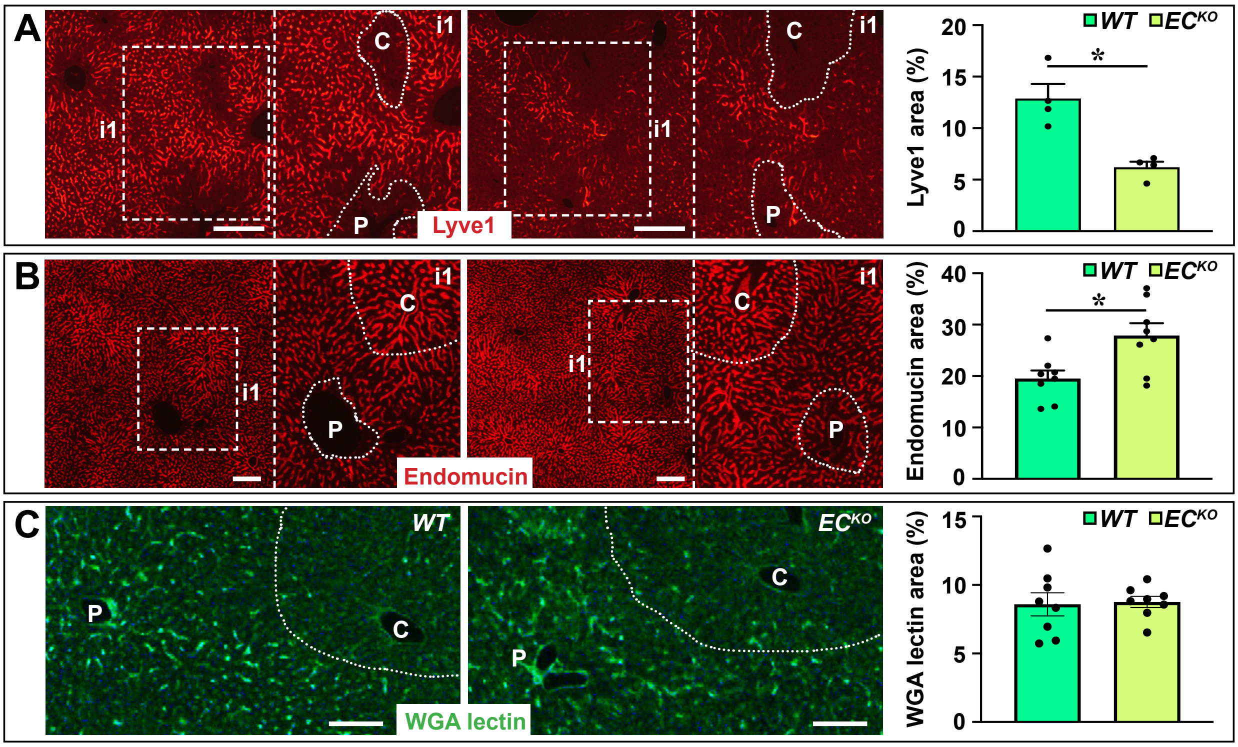

Figure S5. Endothelial *Zeb2* loss does not affect LSEC zonation. (**A,B**) Representative pictures of Lyve1 (*A; n*=4) and Endomucin (*B; n*=8) staining and corresponding quantification (% *vs.* total area). (**C**) Representative pictures for *Wheat Germ Agglutinin* (WGA) lectin binding and corresponding quantification (% *vs.* total area; *n*=8). Data are expressed as mean ± sem. *P<0.05. Zones surrounding the central ('C') and portal ('P') vein are marked by white dotted lines. Scale bars: 100 µm (*C*), 200 µm (*A*,*B*).

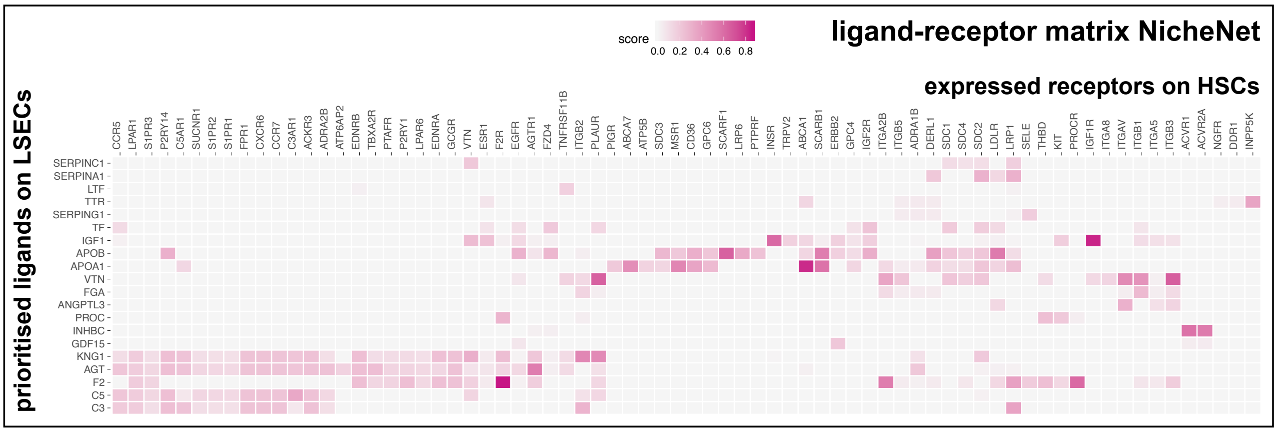

Figure S6. NicheNet analysis upon endothelial-specific *Zeb2*-knock-out. Heat map showing expression of ligands downregulated by *Zeb2* deletion emerging from NicheNet analysis in LSEC source cells and corresponding candidate receptors expressed on *WT* target HSCs.

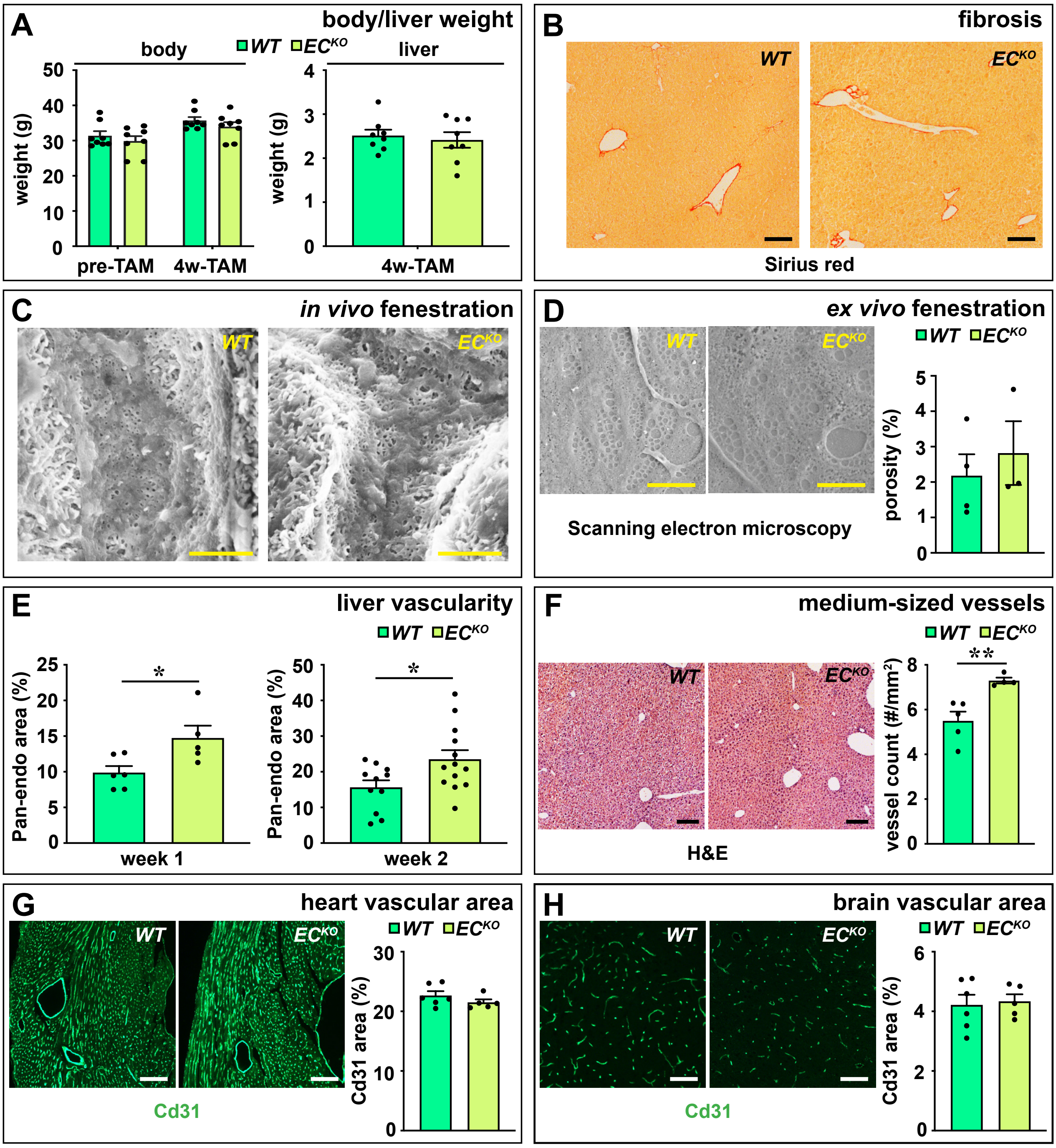

Figure S7. Phenotype of non-challenged EC-specific *Zeb2*-knock-out mice. (A) Body weight and liver weight of mice before ('pre') and after tamoxifen (TAM) treatment. (*n*=7-9). **(B)** Representative images of Sirius red-stained liver sections of *WT* or EC^{KO} livers. **(C)** Scanning EM of liver sections. **(D)** Porosity of liver sinusoidal ECs (LSECs) in liver bulk cultures (*n*=3-4). **(E)** Pan-endo^{+} area 1 (*left; n*=5-6) or 2 (*right; n*=11-13) weeks after the last TAM injection. **(F)** Number of medium-sized vessels counted on H&E-stained sections (*n*=4-5). **(G,H)** Cd31^{+} area in heart (*G*) and brain (*H*) (*n*=5-6). Data represent mean ± sem; **P*<0.05, ***P*<0.01. Scale bars: 2 µm in *C,D*; and 100 µm in *B,F-H*.

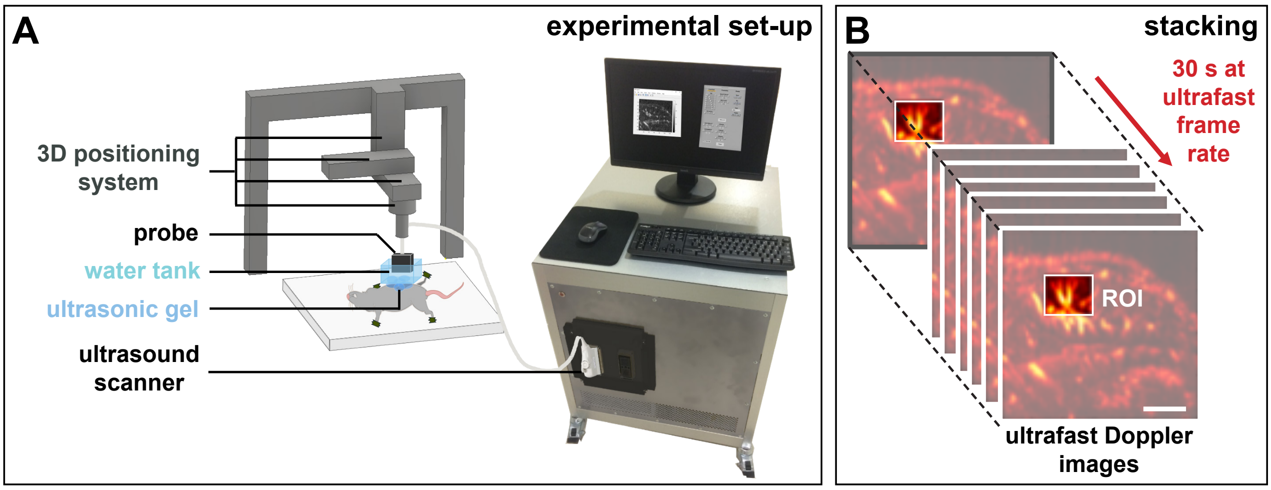

Figure S8. Ultrafast ultrasound imaging of the liver. (A) Schematic representation of the experimental set up for *in vivo* ultrasound imaging of the liver. (**B**) Example of a stack of ultrafast Doppler images of the liver recorded during 30 seconds (s) at ultrafast frame rate. ROI: region of interest. Scale bar: 3 mm.

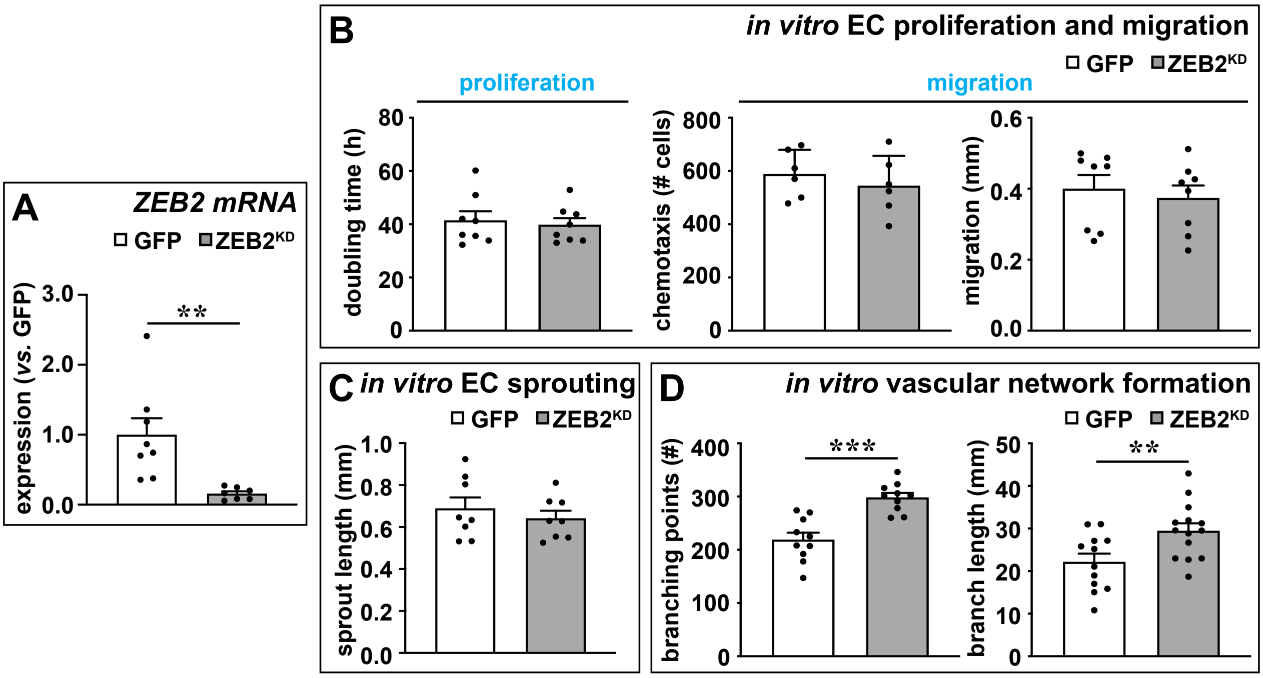

Figure S9. ZEB2 affects tube formation in HUVECs. (A) *ZEB2* mRNA expression in HUVECs transduced with a lentivirus for GFP (control) or shRNA for ZEB2. (n=7-8). **(B)** HUVEC proliferation (n=8) chemotaxis (n=6) and scratch wound closure (n=8). **(C)** Sprout length from HUVEC spheroids (n=8). **(D)** Number of branching points (n=12-14) and branch length in HUVECs grown on Matrigel (n=12-14). Data represent mean ± sem; **P<0.01, ***P<0.001.

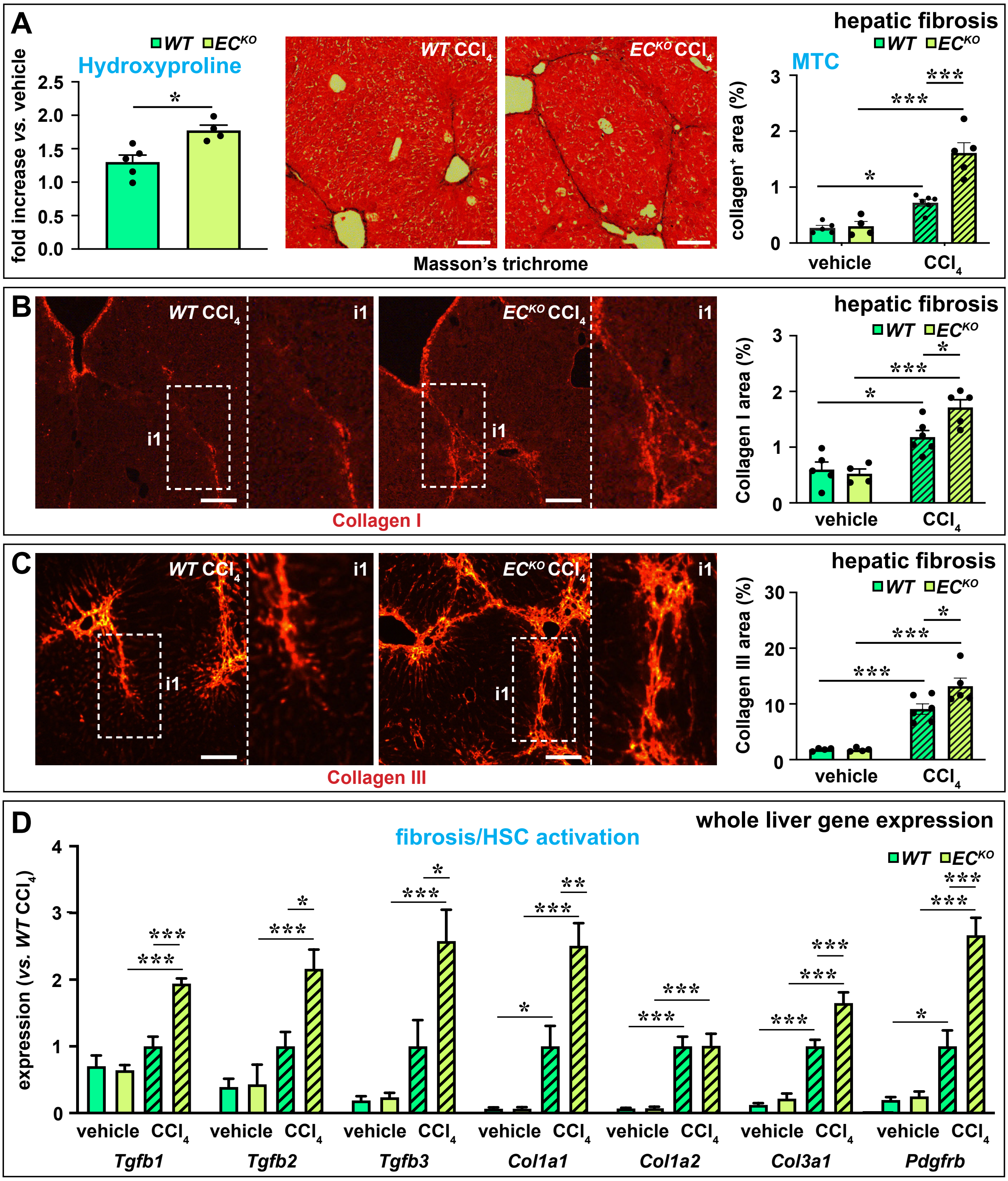

Figure S10. Increased liver fibrosis in EC-specific *Zeb2*-knock-out mice upon chronic CCl_4 challenge. (A) Hydroxyproline levels (*n*=4-5; expressed as fold increase *vs.* vehicle) and collagen$^+$ area on Masson's trichrome staining (*n*=4-6) in livers of mice after 4 weeks of treatment with oil (vehicle) or high-dose CCl_4 and representative pictures. **(B,C)** Collagen type I$^+$ (*B*) and Collagen type III$^+$ (*C*) area in livers of mice treated for 4 weeks with oil (vehicle) or high-dose CCl_4 (*n*=4-6). **(D)** Expression of genes related to fibrosis/hepatic stellate cell (HSC) activation in whole livers after 4 weeks of treatment with oil (vehicle) or high-dose CCl_4 (*n*=4-6). Data represent mean ± sem; **P*<0.05, ***P*<0.01, ****P*<0.001. Scale bars: 100 μm.

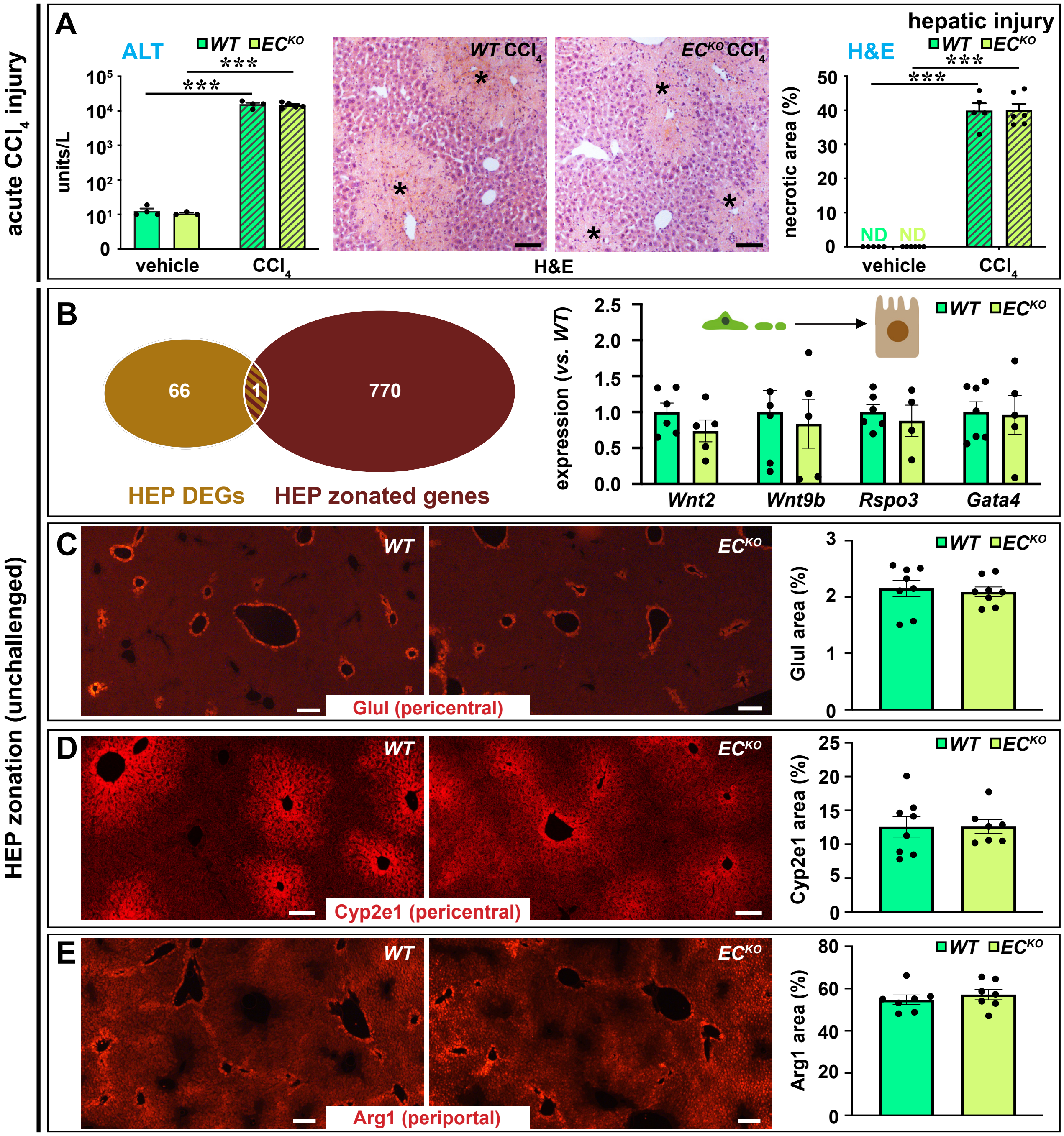

Figure S11. Loss of endothelial *Zeb2* does not affect acute hepatocyte damage or zonation. (A) Plasma alanine transferase (ALT) levels (*n*=3-5) and necrotic area (*n*=5-6) in livers of mice after a single injection of oil (vehicle) or CCl_4 and representative H&E pictures; areas showing necrosis are indicated by asterisks. ND: not detectable. (**B**) Diagram showing minimal overlap between differentially expressed genes (DEGs) in hepatocytes (HEPs) from EC^{KO} livers with genes known to be zonated (*left*) and mRNA expression of angiocrine factors known to regulate HEP zonation in LSECs of EC^{KO} *vs. WT* mice (*right; n*=4-7). (**C-E**) Representative pictures for HEP zonation markers Glutamate ammonia ligase or Glul (*C*), Cytochrome P450 Family 2 Subfamily E Member 1 or Cyp2e1 (*D*) and Arginase1 or Arg1 (*E*) and corresponding quantification (% *vs.* total area; *n*=7-8). Data are expressed as mean ± sem; ****P*<0.001. Scale bars: 100 µm (*A,D*), 200 µm (*C,E*).

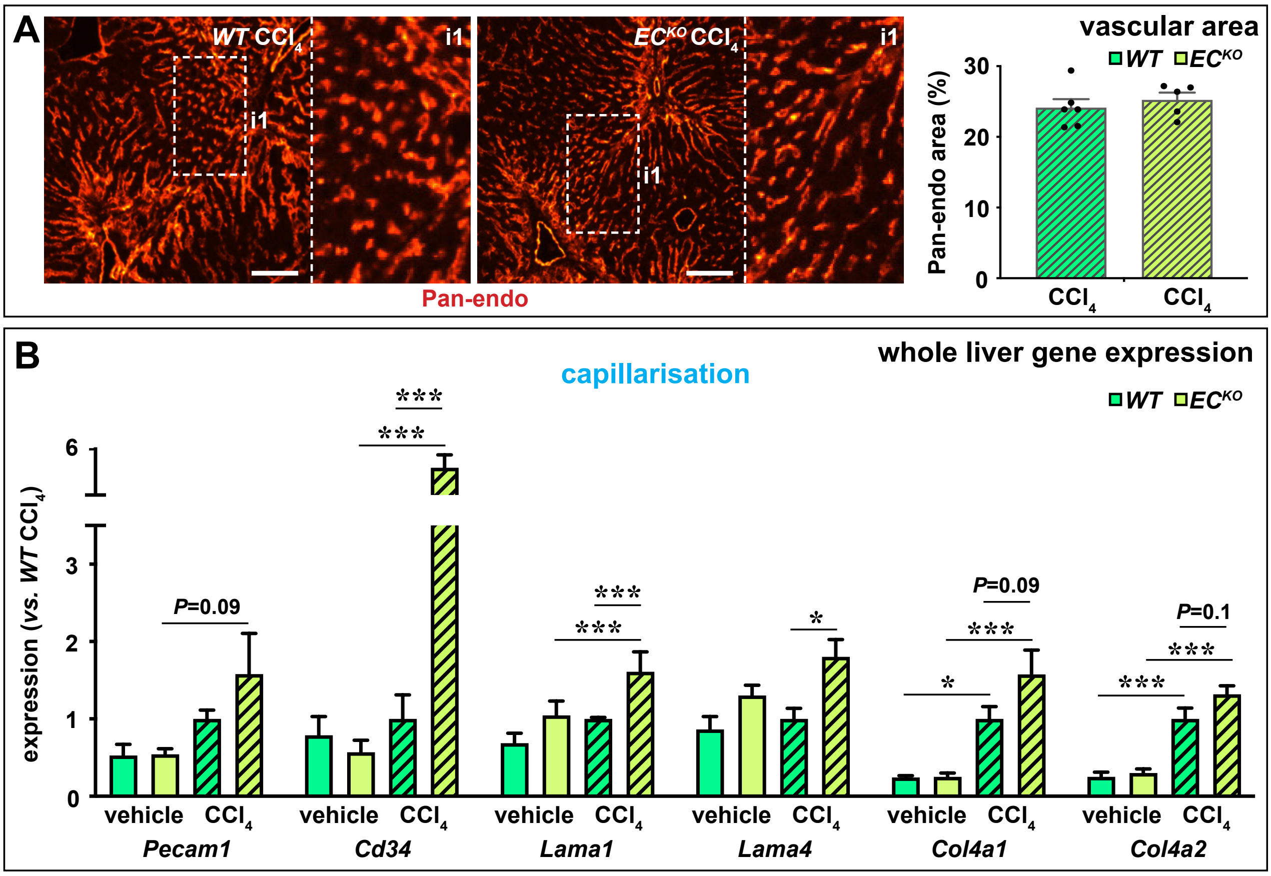

Figure S12. Vascular phenotype of EC-specific *Zeb2*-knock-out mice upon chronic CCl_4 challenge. (A) Pan-endo[+] area in livers of mice treated for 4 weeks with high-dose CCl_4 (*n*=4-9). **(B)** Expression of genes related to capillarisation in whole livers after 4 weeks of treatment with oil (vehicle) or high-dose CCl_4 (*n*=4-6). Data represent mean ± sem; **P*<0.05, ****P*<0.001. Scale bars: 100 μm.

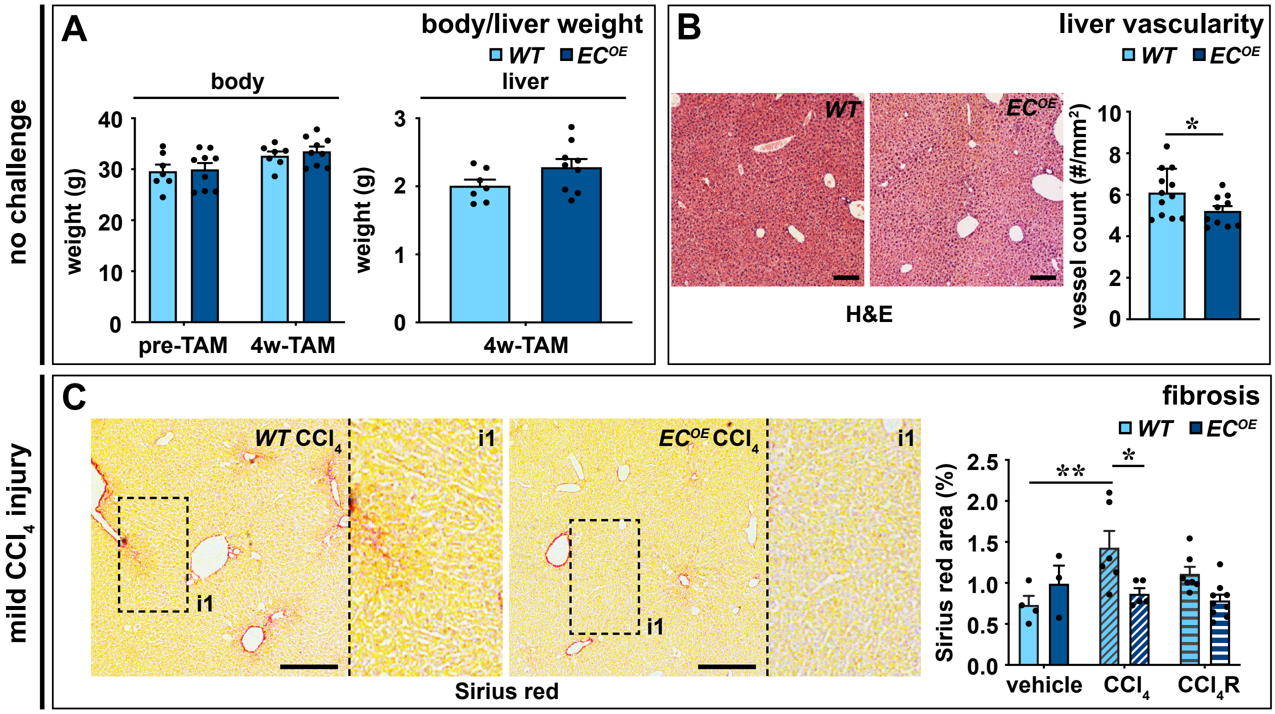

Figure S13. Phenotype of EC-specific *Zeb2*-overexpressing mice unchallenged and upon mild CCl_4 challenge. (A) Body weight and liver weight of wild-type (*WT*) or endothelial cell (EC)-specific *Zeb2*-overexpressing mice (EC^{OE}) before and after last tamoxifen treatment (*n*=5-8). **(B)** Number of medium-sized vessels counted on H&E-stained sections (*n*=10-12). **(C)** Sirius red^{+} area (*n*=4-9) after treatment with oil (vehicle) or CCl_4 24 hours (progression cohort) or 1 week after the injection (regression cohort 'R'). Data represent mean ± sem; **P*<0.05, ***P*<0.01. Scale bars: 100 µm.

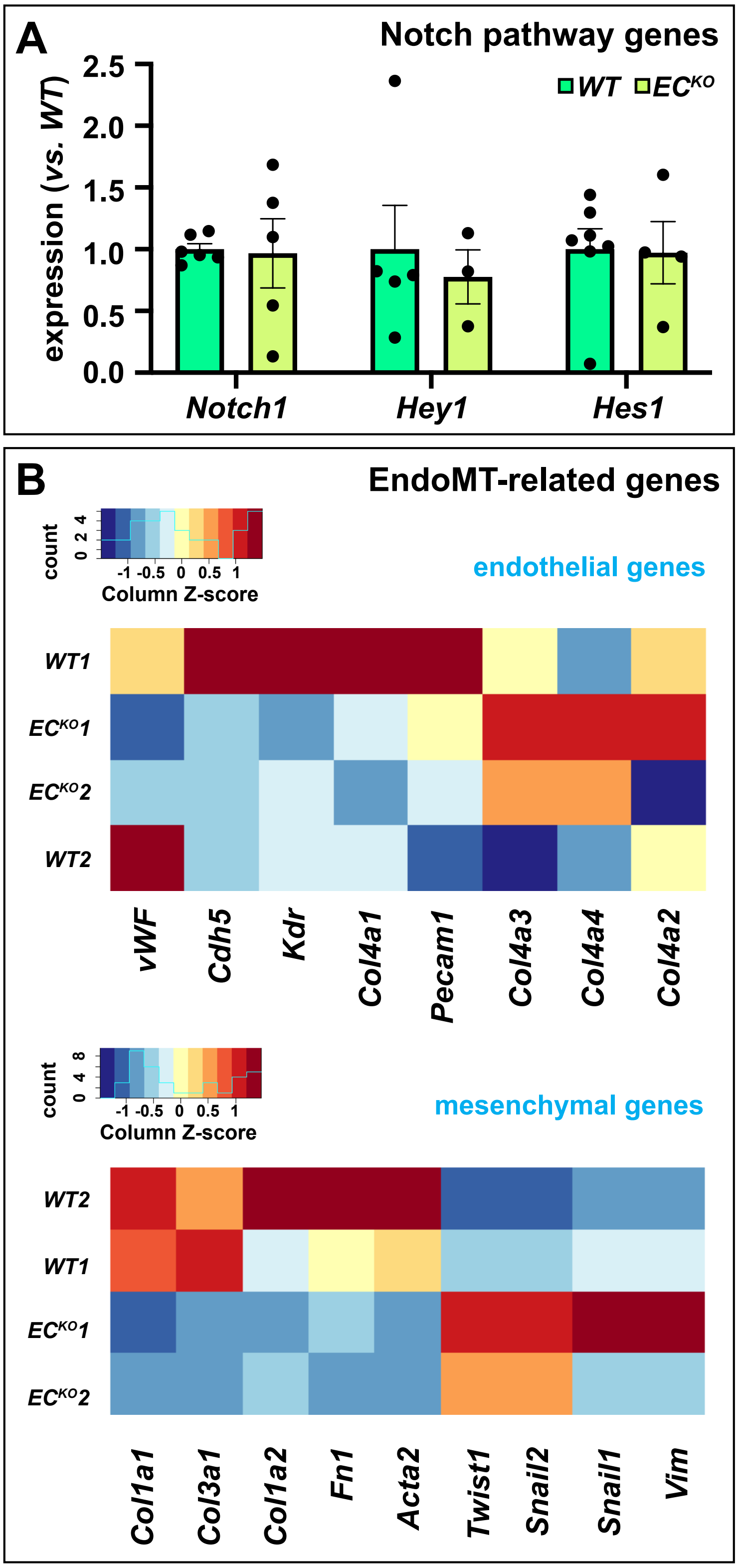

Figure S14. Analysis of EndoMT-related genes and Notch pathway genes upon endothelial-specific *Zeb2*-knock-out. (**A**) Expression of Notch pathway genes in wild-type (*WT*; *n*=5-7) *versus* EC-specific *Zeb2*-knock-out (EC^{KO}) mice (*n*=3-5). Data represent mean ± sem. **(B)** Heat map showing expression of endothelial-to-mesenchymal transition (EndoMT)-related genes in LSECs of *WT* *versus* EC^{KO} mice.

B. SUPPLEMENTARY TABLES

Table S1. Antibodies/reagents for immunofluorescence staining and cell isolation

Target	Ab species	Conjugate	use, dilution	Catalogue number
Mouse α-SMA	rabbit	-	IF 1:4,000	Cell Signaling D4K9N
Mouse Arg1	rabbit	-	IF 1:100	Novus NBP1-32731
BS-I lectin	-	biotin	IF 1:100	Sigma L3759
Mouse Cd31	rabbit	-	IF 1:200	Ab28364
Mouse Cd32	rat	-	IF 1:100	BD553142
Mouse Cd34	rat	PE	FACS 1:80; IF 1:100	BD551387
Mouse Cd45	rat	PE	IF 1:100	BD55308
Mouse Ck19	rat	-	IF 1:200	DSHB TROMA-III-c
Mouse Collagen type I	rabbit	-	IF 1:400	Ab34719
Mouse Collagen type III	rabbit	-	IF 1:400	Ab7778
Mouse Collagen type IV	rabbit	-	IF 1:100	Biorad 2150-1470
Mouse Cyp2e1	rabbit	-	IF 1:100	Novus NBP1-85367
Mouse Desmin	goat	-	IF 1:100	R&D AF3844
Mouse Endomucin	goat	-	IF 1:600	R&D AF4660
Mouse/Human Erg	goat	-	IF 1:100	AB115555
Mouse Erg	rabbit	-	IF 1:200	Ab110639
Mouse F4/80	rat	Alexa-647	FACS 1:80	Invitrogen MF48021
Mouse F4/80	rat	PE	FACS 1:80	BD565410
GFP	chicken	-	IF 1:100	Ab13970
GFP	rabbit	-	IF 1:100	Invitrogen A1122
Mouse Glul	rabbit	-	IF 1:100	Novus NB110-4140
Mouse Meca32	rat	Alexa-770	FACS 1:80	Novus NB10077668AF700
Ki67	rabbit	-	IF 1:1000	Ab15580
Mouse Meca32	rat	biotin	FACS 1:80; IF 1:400	BD550563
Mouse Laminin	rabbit	-	IF 1:100	Sigma L9393
WGA lectin	-	FITC	IF 1:1,000	Sigma L4895
Mouse Lyve1	rabbit	-	IF 1:2,000	Angiobio 11034
Mouse vWF	rabbit	-	IF 1:250	Dako A0082
Human ZEB2	rabbit	-	IF 1:100	Made in house
Isotype control	rat	PE	FACS 1:80	BD553989
Isotype control	rat	APC	FACS 1:80	BD550882
Isotype control	rat	Alexa-700	FACS 1:80	R&D IC006N
Streptavidin-APC	-	APC	FACS 1:80	BD550882
anti-Chicken IgG	donkey	Alexa-488	IF 1:100	Jackson 703-546-155
anti-Rabbit IgG	goat	biotin	IF 1:300	Dako E0432
anti-Rabbit IgG	goat	HRP	IF 1:300	Dako P0448
anti-Goat IgG	rabbit	biotin	IF 1:300	Dako E0466
anti-Goat IgG	rabbit	HRP	IF 1:300	Dako P0449
anti-Rat IgG	rabbit	biotin	IF 1:500	Invitrogen A18919
anti-Rat IgG	rabbit	HRP	IF 1:500	Invitrogen A18915
Streptavidin	-	Alexa-488	IF 1:200	Life tech S11223

Abbreviations: GFP: green fluorescent protein; PE: phycoerythrin; FITC: fluoresceine-isothiocyanate; APC: allophycocyanin; HRP: horse radish peroxidase; IF: immunofluorescence; SMA: Smooth muscle actin; vWF: von Willebrand Factor; BS-I: *Bandeiraea simplicifolia*-I; Erg: ETS-related gene; Ck19: Cytokeratin 19; WGA: *Wheat Germ Agglutinin*; Glul: glutamate ammonium ligase; Cyp2e1: Cytochrome P450 Family 2 Subfamily E Member 1; Arg1: Arginase1.

Table S2. Primer sequences for qRT-PCR

Gene	Forward (5' > 3')	Reverse (5' > 3')
**A. Mouse genes**		
18s	*TCAAGAACGAAAGTCGGAGGTT*	*GGACATCTAAGGGCATCACAG*
Acta2	*CTACGAACTGCCTGACGGG*	*GTTTCGTGGATGCCCGCT*
Agmat	*CCTCTGACCTTGGGTGGAGA*	*GCACCCACATGCACTAAACC*
Akr1d1	*GCTTGCAAAGATGCTGGCTT*	*GAAATACGGGTGGCACTCCA*
Angptl4	*GGGGACCTTAACTGTGCCAA*	*GTTGCCGTGGGATAGAGTGG*
Angptl3	*GCGAACATACAAGTGGCGTG*	*TCGTTGAAGTCCTGTGAGCC*
Apln	*CATAAGGGCCCCATGCCTTT*	*TGGAGTAGAGAGACCGGCAC*
Apoa1	*TTTGAATCCTCCTCCTTGGGC*	*CAGGTTATCCCAGAAGTCCCG*
Apoa5	*GTTGGAGCAAAGGCGTGATG*	*CTTGCTCGAAGCTGCCTTTC*
Apoc3	*GAAACAAAGAGCTGGAGGGAGA*	*TACGTACCATGAGTCCCAAGC*
Bhmt	*CCTCAGAGCTGGATCGAACG*	*CCGTGCAATGTCACAAGCAG*
Bmp1	*GTACGTGGGCTATCTCCAGC*	*TTGGCATCACGTCCATCGAA*
C3	*TGCCCCTTACCCCTTCATTC*	*GCAGTAGCACTAGTAGCTGGG*
C8a	*GCTTGTCTTTGGTGGCATGG*	*ACTGACAGGACACTGCTTGC*
C9	*GGAAGTCTCCCCAGCAGAAC*	*GGACCACTCCTCTCAAGTGC*
Ccl2	*CCCAATGAGTAGGCTGGAGAG*	*GACCCATTCCTTCTTGGGGTC*
Ccl5	*TTGGCATCACGTCCATCGAA*	*AGGGAAGCTATACAGGGTCAGA*
Cd34	*AGCAGTAAGACCACACCAGC*	*AGTTCCAGAGCCTGAAGGGA*
Cepba	*AATGGCAGTGTGCACGTCTA*	*CCCCAGCCGTTAGTGAAGAG*
Chac1	*GCCTATAGTGACAGCCGTGT*	*CATCTTGTCGCTGCCCCTAT*
Clec4g	*AATACAACAAGCTGGGCAGTG*	*TGGACAGTAGGGTGCTCAGA*
Col1a1	*AGCACGTCTGGTTTGGAGAG*	*GACATTAGGCGCAGGAAGGT*
Col1a2	*GGCTCTAGAGGTGAACGTGG*	*CACCAGGGGCACCATTAACT*
Col3a1	*CATACCTGGTACCGGTGGTC*	*CACCGACTTCACCCTTTGGA*
Col4a1	*CAGGACAAAAGGGTGATGCT*	*CCTTTGTACCGTTGCATCCT*
Col4a2	*CATCCGTCGGAGATGAAGAT*	*GTGTTCCTGGGAAACCTGAA*
Col18a1	*CCAAGTTCTCCAGATGCTCAGA*	*CCCACCTCCTCAGCAACATT*
Cps1	*TTTGATTGCTGGGGGACCTG*	*CAATGGCTCTTTGCGGTCAC*
Cxcl12	*TCCAAGAGTACCTGGAGAAAGC*	*GCAGGAAGCGGGGAACTA*
Cxcr6	*TGGAACAAAGCTACTGGGCT*	*TCGTAGTGCCCATCGTACAG*
Cyp2d10	*CATCAGGATGCAGAAAGTACTGG*	*CGCAGGAGTATGGGGAACATA*
Cyp8b1	*TTGCAAATGCTGCCTCAACC*	*TAACAGTCGCACACATGGCT*
Des	*AACTTCCGAGAAACCAGCCC*	*ATCCGAGAGTGGAAGAGGCT*
Egfr	*TGGGTGGCCTCCTCTTCATA*	*GGTGTGAGAGGTTCCACGAG*
F8	*TTTATTGCAGCTGTGGAACG*	*ACAACCCCAGGTGTTCATTT*
Fabp1	*GTCAGCTGTGGAAAGGAAGC*	*GTCTCCAGTTCGCACTCCTC*
Fcgr4	*CAGAGGGCTCATTGGACACA*	*CACGGTGGAAACATGGATGG*
Fgb	*ATGACCATCCACAACGGCAT*	*GATCCGTAGTTACCCAGCCG*
Flt	*TGGCCAGAGGCATGGAGT*	*TCGCAAATCTTCACCACATGG*
Fn1	*TGGAGCCAGGAACCGAGTA*	*TTGGGGTGTGGAAGGGTAAC*
G6pc	*CCGGATCTACCTTGCTGCTC*	*GCATTGTAGATGCCCCGGAT*
Gapdh	*CCGCATCTTCTTGTGCAGT*	*GAATTTGCCGTGAGTGGAGT*
Gata4	*TTTCCTGAGCAAACCAGAGC*	*GCGGAACTGTCACCAAAAAT*
Gdf15	*TGAGTCCCAACTCAACGCC*	*ACCCCAATCTCACCTCTGGA*
Hes1	*TCAACACGACACCGGACAAA*	*ATTCTTGCCCTTCGCCTCTT*
Hey1	*ATGAAGAGAGCTCACCCAGACTAC*	*ATCTGCAAGATCTCAGCTTTTTCT*

Hprt1	*CAGTACAGCCCCAAAATGGT*	*TTGCGCTCATCTTAGGCTTT*
Igf1	*ATGTTCCCCCAGCTGTTTCC*	*ATTCCATTGCGCAGGCTCTA*
Igfbp1	*GGAACGCCATCAGCACCTA*	*GTTGGGCTGCAGCTAATCTCT*
Inhbe	*CCTGGCAACCGAGAGAAAGT*	*CATGGTACAGGTGGTGGGAC*
Itgam	*AGCTTGGCTTTTTCAAGCGG*	*AAAGGCCGTTACTGAGGTGG*
Knca2	*ATGGAGGCTCTGGTACCCAT*	*AGGAAGGAGGCAAGATGCAC*
Krt8	*TCTCCGAGATGAACCGCAAC*	*TTAATGGCCATCTCCCCACG*
Lama1	*ATTTCAGCCCTACCTGCCAC*	*CATGGTACACAGGTTCCCCC*
Lama4	*GGAGAATGTGCACCCTGTGA*	*CTCTCCTGTTGTGTTCCGCT*
Lgmn	*ATTTGCATTGCCCAGTAAGG*	*ACATCTGTGCCGTTAGGTCG*
Ltf	*TGATGACACCCGGAAACCTG*	*CGGTCGCTATGACGTACTCC*
Lyve1	*ACCTGGAAGCCTGTCTCTGA*	*AGGAGCCCTCTCCTTACTGC*
Map1b	*CGCACCGCTTCCTAGACA*	*TCAGGTTTGTGTCCCACGAT*
Mmr	*TATGTTTGGGAGTGCCATCA*	*ATTTGCATTGCCCAGTAAGG*
Msr	*CAATGACAGCATCCCTTCCT*	*CATTTCCCAATTCAAAAGCTG*
Notch1	*TGGATGTCAATGTTCGAGGAC*	*TCTTCTTCACTGTTGCCTGTCTC*
Oaz1	*AGTCAGCGGGATCACAGTCT*	*CCAAGAAAGCTGAAGGTTCG*
Pck1	*TGCGGATCATGACTCGGATG*	*AGGCCCAGTTGTTGACCAAA*
Pdgfb	*GGAGTCGGCATGAATCGCT*	*CATCAAAGGAGCGGATGGAGT*
Pdgfrb	*ACGACCATGGCGATGAGAAA*	*AAGGCATCGGATAAGCCTCG*
Pecam1	*CTGGAAATGATGCAGTAAAACCCA*	*AGCCTTCCGTTCTCTTGGTG*
Pparg	*CAGGCCTCATGAAGAACCTT*	*GGATCCGGCAGTTAAGATCA*
Prodh2	*GTGTGACCATGTCTCCCTGG*	*TGGGCTCTTCGGATCAGGTA*
Rbp4	*GAGTTTGGCTCCACCGAGAC*	*ACCCACTCCATCTCACCCC*
Reep6	*GCGGAAGAGCATTGGACCTA*	*CTTGGGGTCCAGTTCCAGAG*
Rgcc	*ATTGCCGATCTGGACAGGAC*	*GTGAACCAAGAATGGCCAGG*
Rspo3	*TGCCCAGAAGGGTTAGAAGC*	*CCGTGTTTCAGTCCCCCTTT*
Serpina1a	*GACTGCCACCTCACGAGAAT*	*TGCCCAAAAGTGGTGTAGCA*
Serpina1b	*AAGGTGCCCATGATGATGCT*	*TGCCCGCGTAATCCATCAG*
Serpinc1	*GACTGCCACCTCACGAGAAT*	*TGCCCAAAAGTGGTGTAGCA*
Slco1b2	*TGATCGGACCAATCCTTGGC*	*AACCCAACGAGCATCCTGAG*
Sod3	*ACTTCACCAGAGGGAAAGAGC*	*CTGGACTCCCCTGGATTTGAC*
Stab1	*GGATTGCCAAGCCTTGAACA*	*GCAACCGCGTTTTGTGATG*
Stab2	*ATGCACTTGCCAGAAAGGTT*	*ATGCACTTGCCAGAAAGGTT*
Steap4	*GGCTCTCCAGTCAGGAACAC*	*GGTGAGCCCAAGAGTACGAG*
Tgfb1	*ATGCTAAAGAGGTCACCCGC*	*TGCTTCCCGAATGTCTGACG*
Tgfb2	*CAGTGGGAAGACCCCACATC*	*TGTAAAGAGGGCGAAGGCAG*
Tgfb3	*GATCACCACAACCCACACCT*	*ATAAAGGGGGCGTACACAGC*
Tie1	*GCATGGGACGGCCTCC*	*TCCCTGTGAATGAACTGCTTCTC*
Tmprss6	*CCAGGCCATTGATCCAACCT*	*GAACAGGGGAGCTGGAAACA*
Wnt2	*GCCAACGAAAAATGACCTCGT*	*TCGGGAAGTCAAGTTGCACA*
Wnt9b	*GGCCCAAGAGAGGAAGCAAG*	*CCTGACACACCATGGCACTT*
Ywhaz	*GCTGGTGATGACAAGAAAGGAAT*	*GCGTGCTGTCTTTGTACGAC*
Zeb1	*CTGCTCCCTGTGCAGTTACA*	*GTGCACTTGAACTTGCGGTT*
Zeb2	*GGACTGCAAGACGGAAGACA*	*TTGAGTCGGTGGTCAAGCTC*
**B. Human genes**		
GAPDH	*TGGTATCGTGGAAGGACTCATGAC*	*ATGCCAGTGAGCTTCCCGTTCAGC*
ZEB2	*ATGCTTTTGCCCAACTGCTG*	*CTCGTGGCGGTACTTGATGT*

C. EXTENDED METHODS

Mice and human tissues/cells

Mouse experiments were approved by the KU Leuven Animal Ethics Committee (Ethics Committee Dossier 148/2010, 022/2011, 169/2014, 208/2017 and 121/2019) or the VUB ethics Committee (VUB-18-212-2) and were performed in accordance with the Committee's guidelines and those from directive 2010/63/EU of the European Parliament on the protection of animals used for scientific purposes. Mice were housed in filter top cages with wood bedding and cocoons as enrichment under standard conditions with 12-hour light/dark cycles. Mice had *ad libitum* access to regular chow (Ssniff) and water. All interventions were done during the light cycle. Unless stated otherwise, male mice were used that were 8 weeks of age at the start of the experiment. Blood was drawn via the heart and mice were euthanised by exsanguination under ketamine (75 µg/g i.p.) and xylazine (5 µg/g i.p.) anaesthesia. Anaesthesia depth was checked by toe pinch and, if necessary, mice received another i.p. injection with 7.5 µg/g ketamine/0.5 µg/g xylazine. Four different mouse lines were used: (1) endothelial cell (EC) reporter mice specifically expressing green fluorescent protein (GFP) in blood-vascular but not lymphatic ECs under the *Tie2* promoter ('*Tie2-GFP'* on an FVB background);[1] (2) *Zeb2* knock-in reporter mice expressing a Zeb2-enhanced (e)GFP fusion protein under control of the endogenous *Zeb2* promoter ('*Zeb2-eGFP'* on an ICR/C57Bl6 background);[2] (3) endothelial-specific *Zeb2* knock-out mice ('EC^{KO}') generated by inter-crossing the tamoxifen-inducible $Cdh5\text{-}Cre^{ERT2}$ driver line (on a C57Bl6 background)[3] with $Zeb2^{fl/fl}$ mice (on a 129Sv/CD1 background) carrying a *Zeb2 exon 7* flanked by *lox*P sites[4] (Fig. S1A,left; all supplementary figures are designated 'S'); (4) endothelial-specific *Zeb2*-overexpressor mice ('EC^{OE}') generated by inter-crossing the $Cdh5\text{-}Cre^{ERT2}$ driver line with $ROSA26\text{-}Zeb2^{tg/tg}$ mice (on a 129Sv/CD1 background) which carry on both alleles a floxed STOP codon before the *Zeb2* gene under the *Rosa26* promoter in the *Rosa* locus and an *ires-eGFP* cassette in frame with the *Zeb2* gene (Fig. S1A,right).[5] For EC^{KO} and EC^{OE} mice, tamoxifen-treated *Cre*-negative littermates were used as wild-type (*WT*) controls. To enable visualisation and quantification of Cre-mediated recombination, EC^{KO} mice were crossed onto an R26R CAG-boosted eGFP (RCE) reporter background (harbouring a floxed STOP codon before the *GFP* gene under the *CAG* promoter in the *Rosa26* locus; Fig. S1A,left).[6] To induce Cre-mediated recombination, mice were intraperitoneally (i.p.) injected with 2 mg tamoxifen (Sigma) in 100 µL sunflower oil (Sigma) for 5 consecutive days. Recombination efficiency (expressed as the fraction of ECs expressing eGFP) was ~90% and similar across organs and across the zonated liver vascular bed (Fig. S1B,C).

Human liver biopsies used for immunostaining were collected in Gey's buffer after obtaining informed consent from the donors (patients undergoing elective cholecystectomy at the University Hospital UZ Leuven). Biopsies were fixed overnight in 4% paraformaldehyde (PFA), embedded in paraffin and sectioned for immunofluorescence (IF) staining (primary and secondary antibodies used for IF staining are listed in Table S1). Biopsies were stained with Sirius Red and none of the biopsies showed fibrosis. Human umbilical vein ECs (HUVECs) were isolated as previously described[7] from umbilical cords of babies delivered by Caesarean section after full-term pregnancy with informed consent from the mother. The use of human biopsies, umbilical cords and human cells was approved by the Ethics Committee of University Hospitals Leuven (No B32220152525871) and experiments were performed in accordance with the Committee's guidelines and the principles of the Declaration of Helsinki.

Maintenance and hepatotoxic models

To study the effect of endothelial Zeb2 on (vascular) maintenance, tamoxifen-treated mice were euthanised under anaesthesia as described above at 1, 2, or 4 weeks after the last tamoxifen injection (Fig. S1D). Unless indicated otherwise, mRNA expression changes are shown at 2 weeks post-tamoxifen and protein expression or structural changes at 4 weeks post-tamoxifen. To study the response to acute liver injury, mice received one i.p. injection with high-dose CCl_4 (0.6 µL/g in mineral oil) or with mineral oil alone as control (vehicle) and were sacrificed 24 hours later (Fig. S1E). To study the effect of mild fibrosis, mice were injected with low-dose CCl_4 (0.2 µL/g in mineral oil) or vehicle 3 times with one day in between and were sacrificed 24 hours (progression cohort) or 1 week (regression or 'R' cohort) after the last injection (Fig. S1F). For chronic (septal) fibrosis, mice received high-dose CCl_4 (0.6 µL/g in mineral oil) or vehicle 3 times per week (Monday-Wednesday-Friday) for 4 weeks and were sacrificed 24 hours (progression cohort) or 1 week (regression or 'R' cohort) after the last injection (Fig. S1G). For (immuno)histological analyses, mice were euthanised under anaesthesia as described above, livers were subsequently perfusion-fixed with zinc-formalin (Sigma) and processed for paraffin sectioning. For other analyses, tissues were isolated, snap-frozen and stored at -80°C until further use.

Cell isolation and gene profiling

To study the organ-specific *Zeb2* expression, GFP^+ ECs were isolated from *Tie2-GFP* hearts, brains and livers (yielding > 95% pure populations consisting for > 99% of microvascular ECs)[8, 9] and comparative gene expression was performed by quantitative (q) real-time (RT)-PCR. To establish primary cultures for fenestrae analysis, livers were digested and plated in EGM2 medium supplemented with EGM2-MV (containing 0.5 ng/mL VEGF-A; Lonza) onto gelatin-coated cell culture vessels, cells were allowed to settle for 36 hours, washed and fixed with 2.5% glutaraldehyde in 0.1 M sodium cacodylate buffer. To simultaneously isolate the four main hepatic cell types, EC^{KO} and their *WT* (*Cre*-negative) littermates were perfused via the portal vein for 5 minutes with SC-1 solution (8 g/L NaCl, 400 mg/L KCl, 75.5 mg/L $NaH_2PO_4.H_2O$, 120.45 mg/L Na_2HPO_4, 2.38 g/L HEPES, 350 mg/L $NaHCO_3$, 190 mg/L EGTA, 900 mg/L glucose, pH 7.3), followed by 5 minutes 0.04% pronase E (Merck) and 5 minutes 0.02% collagenase P (Boeringer-Manheim) both in 8 g/L NaCl, 400 mg/L KCl, 75.5 mg/L $NaH_2PO_4.H_2O$, 120.45 mg/L Na_2HPO_4, 2.38 g/L HEPES, 350 mg/L $NaHCO_3$, 560 mg/L $CaCl_2.2H_2O$, pH 7.3). The liver was excised and incubated at 37°C for 15 minutes in a solution containing 0.03% collagenase P, 0.03% pronase E and 0.001% DNase (Grade II, Boeringer-Manheim). The resulting suspension was filtered through a 100 µm strainer and centrifuged 2 times for 2 minutes at 50 g to separate the hepatocytes (HEPs) from the non-parenchymal cell (NPC) fraction. Hepatocytes were immediately snap-frozen after centrifugation. Red blood cells were removed from the NPC fraction by incubation with red blood cell lysis buffer (Miltenyi Biotec) for 3 minutes, followed by washing in GBSS-B (70 mg/L KCl, 210 mg/L $MgCl_2.6H_2O$, 70 mg/L $MgSO_4.7H_2O$, 120 mg/L Na_2HPO_4, 30 mg/L KH_2PO_4, 990 mg/L glucose, 227 mg/L $NaHCO_3$, 225 mg/L $CaCl_2.2H_2O$, pH 7.3). The resulting cell pellet was resuspended in a 3:1 mix of SC-1 and DNase solution and the suspension was filtered through a 40 µm mesh. The cell suspension was subsequently stained for Pan-endo and F4/80 to enable the sorting of UV^+ HSCs, UV^- $F4/80^+$ Kupffer cells and UV^-Pan-$endo^+$ ECs on a FACS Aria-II sorter. Antibodies used for FACS are listed in Table S1.

For RNA sequencing, RNA was isolated from ECs, HSCs, Kupffer cells and hepatocytes from *WT* and EC^{KO} mice (*n*=2 per cell type and genotype) using Reliaprep (Promega). RNA samples were quality controlled and prepared according to the Smart-seq2 method.[10] In brief, polyA^{+} RNA was reverse transcribed using an oligo(dT) primer. Template switching by reverse transcriptase was achieved using an LNA-containing TSO oligo. The reverse transcribed cDNA was pre-amplified with primers for 18 cycles followed by clean-up. Tagmentation was performed on 500 pg of the pre-amplified cDNA with Tn5 followed by gap repair. The tagmented library was extended with Illumina adaptor sequences by PCR for 14 cycles and purified. The resulting sequencing library was measured on a Bioanalyser and equimolar loaded onto a flow cell and sequenced according to the Illumina TruSeq v3 protocol on the HiSeq2500 with a single read 50 bp and dual 9 bp indices. Illumina adapter sequences and poly-A stretches were trimmed from the reads.[11] The remaining sequences were aligned to the mouse GRCm38 reference sequence using HISAT2 (version 2.1.0).[12] Transcript abundance level (transcript count) was generated using HTSeq (version 0.9.1) based on the ENSEMBL 84 gene annotation.[13] The transcript counts were further processed using R software environment for statistical computing and graphics (version 3.4.0). Data normalisation, removal of batch effect and other variance was performed using EDASeq R and RUVSeq packages.[14, 15] Differential expression analysis was performed using the edgeR R package,[16] applying the negative binomial general linear model (GLM) approach. Differentially expressed genes with false discovery rate (FDR) < 0.05, Benjamini-Hochberg multiple testing correction and expression level in *WT* samples of > 1 counts per million (CPM) were retained and used for further processing (*i.e.*, principal component analysis, cluster analysis, Volcano plots, heat maps, Gene Enrichment analysis by Enrichr[17] and ligand-target prediction analysis by NicheNet (using an open source R implementation; https://github.com/saeyslab/nichenetr).[18] The RNA sequencing datasets generated for this study are available in the NCBI GEO repository (https://www.ncbi.nlm.nih.gov/geo/), under series number GSE150699. To obtain RNA from whole liver tissue, the liver was homogenised in TRIZol and RNA was isolated according to manufacturer's instructions. For quantitative qRT-PCR, cDNA was made using the GoScript™ reverse transcription system (Promega) according to the manufacturer's protocol. QRT-PCR was performed with the ABI system using Sybr green (ABI). *Gapdh* was used as reference gene after validation (Note S1; for primer sequences, see Table S2).

***In vitro* angiogenesis assays and lentivirus-mediated knock-down**

HUVECs were cultured in EBM2 medium supplemented with EGM2-MV (Lonza) in gelatin-coated culture flasks. Plasmids containing *ZEB2*-shRNA or GFP (control) in a lentiviral backbone were purchased from Sigma and lentiviruses were produced in human embryonic kidney (HEK)293 cells. Obtained viruses were titrated on HUVECs and the lowest concentration leading to 100% transduction efficiency was used in subsequent experiments. Cells were transduced with viruses, medium was changed, 1 and 3 days after transduction and on day 6 cells were either lysed in TRIzol for RNA isolation or passaged for functional analyses.

For proliferation assays, 5,000 cells were plated per well on gelatin-coated 24-well plates, cells were counted 6 days later and doubling time was calculated. For tube formation assays, 50,000 cells were plated on Matrigel-coated 24-well plate and tube formation was assessed 24 hours later. Branch length was quantified using the angiogenesis analyser plug-in for Image J (NIH). For scratch wound assays, 50,000 cells were plated in a 24-well plate, and 24 hours later when confluence was reached,

a standardised scratch was made and the gap distance between the cells was measured immediately after scratching and again 24 hours later. To assess chemotactic migration, 25,000 cells were seeded in starvation medium onto collagen type I-coated Boyden chambers with 8 micron pores (Costar) placed into 24-wells filled with EBM2-EGM2-MV medium and cells were allowed to migrate for 24 hours, as described.[19] Cells were stained with Wright-Giemsa solution (Sigma) and migrated cells were counted. To evaluate sprouting, spheroids were made by incubating 600 HUVECs per spheroid for 24 hours in hanging droplets containing 0.24 g/L methylcellulose in EBM2-EGM2-MV medium. Spheroids were embedded in 2 mg/mL collagen (Corning) and collagen gels were covered with EBM2-EGM2-MV medium. Sprouting was analysed 24 hours later.

Scanning electron microscopy

To perform morphometric analysis on vascular corrosion casts, mice were euthanised under anaesthesia as described above and livers were consecutively perfused with 50 U/mL heparin in PBS, 2.5% glutaraldehyde in PBS and VasQtec resin (VasQtec Zurich, Switzerland; prepared according to manufacturer's instructions). Livers were isolated and kept in 2.5% glutaraldehyde in PBS at 40°C to allow the resin to harden and livers were subsequently saponified in 5% KOH at 40°C for 2-5 days, casts were dried and sputter-coated with gold and images were recorded on a JEOL scanning electron microscope at 0.8 K magnification (JEOL Europe BV). Pillars, the ultrastructural hallmark of intussusceptive angiogenesis, were identified and counted. Sinusoidal diameters were measured in ImageJ. To document fenestrae *in vivo*, mice were perfused via the portal vein with PBS containing 50 U/mL heparin and 2.5% glutaraldehyde in 0.1 M sodium cacodylate buffer (pH 7.3), livers were dissected and fixed overnight in 2.5% glutaraldehyde, buffered with 0.05 M sodium cacodylate (pH 7.3). Liver slices were post-fixed in 2% OsO_4 (buffered with 0.05 M sodium cacodylate, pH 7.3) dehydrated in a graded ethanol series and hexamethyldisilazane before slices were dried, sputter-coated with chrome and pictures were recorded on a Zeiss Sigma VP scanning electron microscope using the variable pressure mode at 24 K magnification. To analyse fenestrae *ex vivo*, digested livers were cultured and bulk cultures were fixed 36 hours later with 2.5% glutaraldehyde (pH 7.3), buffered with 0.05 M sodium cacodylate. Cell cultures were post-fixed in 2% OsO_4 (buffered with 0.05 M sodium cacodylate, pH 7.3) dehydrated in a graded ethanol series followed by hexamethyldisilazane and cell cultures were dried, sputter-coated with chrome and pictures were recorded on a Zeiss Sigma VP scanning electron microscope at 12.5 K magnification. Porosity was determined by calculating the cumulative area taken up by fenestrae relative to the cytoplasmic area. Bulk cultures were used and supplemented with 0.5 ng/mL VEGF-A in order to preserve fenestrae.[20]

Ultrafast ultrasound imaging

Ultrafast ultrasound acquisition was performed on the left (lateral) liver lobe at 2 or 4 weeks after the last tamoxifen injection on a research ultrafast platform (INSERM "Biomedical Ultrasound" ART) comprising an ultrasound acquisition board (128 channels, 62.5 MHz, Verasonics, Vantage 256) and a 15 MHz ultrasonic probe (pitch 0.11 mm, 128 elements, Vermon, France) mounted on 4 motors (3 translation and 1 rotation, Pi, Germany) (Fig. S8). The scanner was driven by a real-time Doppler acquisition software platform (INSERM "Biomedical Ultrasound" ART and Iconeus, Paris, France). Mice were anaesthetised with ketamine (75 µg/g i.p.) and medethomide (1 µg/g i.p.). Anaesthesia depth was checked by toe pinch and, if necessary, mice received another i.p. injection with 7.5 µg/g

ketamine and 0.1 ug/g medothomide. A 3-dimensional printed water tank consisting of TPX polymethylpentene film was placed on the mouse abdomen with transmission gel between the mouse skin and the film and the tank was filled with water. The probe was placed in the water tank in contact with the liver and several planes of the lateral left liver lobe were acquired every 0.5 mm. The animals received subcutaneously atipamezole (1 μg/g) to reverse the anaesthesia, the average imaging time was 45 minutes per mouse. The ultrasensitive ultrasound sequence consisted of 11 compounded plane waves (angles from -10° to 10°) designed to acquire a full 30 seconds at a continuous framerate set to 500 Hz. A singular value decomposition clutter filter was applied on blocks of 200 frames (400 ms) to separate blood signal from tissue signal[21] by removing the 60 largest singular values. In order to filter different flow velocities, additional bandpass frequency filters were applied on the 400 ms subsets of data acquisition, giving access to blood volume movies corresponding to three different velocity ranges (2 to 4 mm/s, 4 to 6 mm/s and 6 mm/s and upper).[22] Those high framerate power-Doppler movies were computed by integrating the energy of windows of 20 consecutive filtered ultrafast frames (40 ms). Breathing motion (which can induce significant out of plane movements and artefacts) was eliminated by performing frame classifications on the high frame-rate power-Doppler movies. For this purpose, a k-means clustering algorithm (2 clusters, correlation distance metric, 100 replicates) was applied to the power-Doppler movies in order to remove the outlier images. The remaining frames were then median-averaged to construct one power Doppler image per band-speed. Regions of interest (ROI) were manually drawn based on the anatomical and vascular landmarks of the liver and the median value of each ROI was estimated for the different bands.

Morphometric analysis and assessment of liver fibrosis and function

For histology, mice were euthanised under anaesthesia as described above and perfusion-fixed via the heart with zinc-formalin (Sigma), the left liver lobe, heart and brain were dissected out, fixed overnight in zinc-formalin, dehydrated, embedded in paraffin and 7 μm sections were prepared. To assess general morphology and liver damage, sections were stained with haematoxylin and eosin (H&E) and collagen was stained using Sirius red, Masson's trichrome (Sigma HT15) or antibodies against fibrillar Collagen types I and III. To analyse the zonated architecture of the liver vasculature, we used antibodies against cytokeratin (Ck)19 and Endomucin to identify portal triads and central veins,[23] respectively. To further characterise the reaction to liver repair/damage, sections were stained with antibodies against α-smooth muscle actin (αSMA), Desmin and the inflammatory marker Cd45. To characterise endothelial changes, IF stainings were performed for Pan-endo, Cd31, Cd32, Cd34, vWF, Laminin, Lyve1, Endomucin, *Wheat Germ Agglutinin* (WGA) lectin and Collagen type IV. To assess Cre-mediated recombination in EC^{KO} mice or demonstrate endothelial expression of Zeb2 in *Zeb2-eGFP* reporter mice, livers were co-stained for eGFP and ETS-related gene (Erg). Alternatively, recombination was assessed by FACS on monocellular liver suspensions stained for Pan-endo. To analyse recombination in different zones, recombined ECs were counted on serial cross-sections stained for Erg/eGFP and pericentral hepatocyte zonation marker Cytochrome P450 Family 2 Subfamily E member 1 (Cyp2e1). Hepatocyte zonation was analysed by IF staining using antibodies against pericentral markers Cyp2e1 or Glutamate ammonia ligase (Glul) and periportal/midzonal marker Arginase1 (Arg1). Antibodies were tested using a negative control (no primary antibody) and *WT* healthy mouse liver sections as positive control where we compare the expression pattern with stainings found on the manufacturers' websites and in literature; for antibodies and concentrations

see Table S1. Where necessary, amplification was performed using a Cy3- or fluorescein-tyramide kit according to the manufacturer's instructions (Perkin Elmer). To demonstrate endothelial Zeb2 expression on human liver paraffin sections, these were co-stained for ZEB2 and Erg (Table S1). Microscopy images were analysed in Image J and FACS data were analysed with FACS DIVA software. To assess fibrosis by hydroxyproline content, livers were hydrolysed in 6 M HCl at 110°C overnight. Liver homogenates were dried and dissolved in 50% isopropanol. Hydroxyproline was detected after oxidation by chloramine T using Ehrlich reagent. To estimate liver function, plasma alanine transferase (ALT) was measured using a Spotchem EZ system analyser (Arkray) and GPT/ALT strips (Menarini diagnostics).

Statistics

Data are expressed as mean ± standard error of the mean (sem), unless stated otherwise. Student's *t*-test was used to compare 2 groups. One-way ANOVA with Bonferroni post-hoc test was used to compare > 2 groups. $P<0.05$ was considered statistically significant. Graphpad Prism 8 was used for statistical analysis.

D. SUPPLEMENTARY NOTES

Note S1. We confirmed that expression of *Gapdh* expression is stable (by measuring its expression using 4 other housekeeping genes as a reference). First, under unchallenged conditions (qRT-PCR data reported in Fig. 2B,C; S11B and S14A), its expression was stable between genotypes in LSECs as well as in HSCs. Second, *Gapdh* expression was also stable upon induction of fibrosis.

**Reference gene**	**normalised *Gapdh* expression**			
	LSECs		HSCs	
	WT (*n*=5-7)	*EC*^{KO} (*n*=4-5)	*WT* (*n*=9-10)	*EC*^{KO} (*n*=7-10)
18s	**1.0 ± 0.1**	1.0 ± 0.3	**1.0 ± 0.2**	1.2 ± 0.2
Hprt1	**1.0 ± 0.1**	1.0 ± 0.3	**1.0 ± 0.2**	0.8 ± 0.1
Oaz1	**1.0 ± 0.1**	0.9 ± 0.3	**1.0 ± 0.3**	0.9 ± 0.1
Ywhaz	**1.0 ± 0.1**	0.9 ± 0.1	**1.0 ± 0.1**	1.0 ± 0.1
	WT		*EC*^{KO}	
	vehicle (*n*=4-5)	CCl_4 (*n*=4-6)	vehicle (*n*=4-6)	CCl_4 (*n*=4-5)
18s	0.8 ± 0.1	**1.0 ± 0.3**	1.1 ± 0.2	1.2 ± 0.1
Hprt1	1.1 ± 0.2	**1.0 ± 0.2**	1.0 ± 0.2	0.9 ± 0.1
Oaz1	1.2 ± 0.1	**1.0 ± 0.0**	1.2 ± 0.1	1.1 ± 0.1
Ywhaz	0.9 ± 0.1	**1.0 ± 0.1**	1.0 ± 0.1	1.0 ± 0.1

Data are expressed as mean ± sem, normalised to the condition indicated in **bold**.

Note S2. As the sorted EC population mainly (> 99%) represents LSECs, we consider that the expression detected largely reflects that in LSECs and therefore labelled the EC population in this results section and corresponding figures as ‘LSECs’ rather than ‘ECs’.

Note S3. The population sizes of each of the three non-parenchymal cell types that were simultaneously sorted were the same between genotypes. The number of hepatocytes counted on H&E-stained liver cross-sections did not reveal differences between the genotypes. Hence, the observed genotypic differences were not indirectly related to a difference in cell numbers.

**cell type**	**fraction (%)**	
	WT (*n*=7)	*EC*^{KO} (*n*=5)
**LSECs**	43 ± 2	41 ± 5
**HSCs**	3 ± 1	4 ± 1
**KCs**	20 ± 2	19 ± 1
	WT (*n*=8)	*EC*^{KO} (*n*=8)
**HEPs**	58 ± 1	58 ± 1

Data are percentages of non-parenchymal cells (LSECs, HSCs and KCs) or all liver cells (HEPs) and expressed as mean ± sem.

Note S4. The expression of multiple genes known to have a zonated expression pattern in LSECs were not altered according to RNA sequencing (including *Kit*, *Lgals1*, *Efnb2*, *Jak1*, *Thbd*, *Fabp4*, *Dll4*, *Cdh13*, *Ltbp4*, *Chst2*, *Pear1* and many others).[24] This gives additional support to the notion that LSEC zonation was not affected by endothelial *Zeb2* loss.

Note S5. The functional importance of the altered LSEC-HSC communication through LSEC ligands Gdf15, Ltf and Igf1 was shown by downregulation of a large panel of their downstream target genes in HSCs.

**Ligand in LSECs**	**Downstream target genes in HSCs**
Gdf15	*Apoc3*, *Ccl2*, *G6pc*, *Krt8*, *Pck1*
Ltf	*Cxcr6*, *Tmprss6*
Igf1	*Bhmt*, *C9*, *Slco1b2*

Note S6. While the vast majority of the studies with EC^{KO} mice were performed with males, some important findings were confirmed in females. A similar increase in Pan-endo^{+} area fraction was observed in EC^{KO} females compared to their *WT* littermates 4 weeks post-tamoxifen (relative area expressed as % *vs.* total area: 25.0 ± 0.7 in EC^{KO} *vs.* 20.1 ± 0.5 in *WT*; *n*=3-5; P<0.01). A similar increase in the number of medium-sized vessels was observed in EC^{KO} females compared to their *WT* littermates 4 weeks post-tamoxifen (number/area in mm^2: 6.6 ± 0.2 in EC^{KO} *vs.* 5.4 ± 0.5 in *WT*; *n*=5-7; P<0.05).

Note S7. Multiple genes known to have a zonated expression pattern in HEPs were not altered in their expression level determined by RNA sequencing (including genes involved in the Wnt signalling pathway, *e.g.*, *Axin2, Axin1, Fzd4, Fzd5, Fzd8, Ctnnb1*, *Apc*, *Lef1* and many others).[25, 26] This gives additional support to the notion that hepatocyte zonation was not affected by endothelial *Zeb2* loss.

Note S8. Increased fibrosis upon exposure of EC^{KO} mice to low-dose CCl_4 was also accompanied by increased signs of capillarisation as evidenced by the increased fractional area of continuous EC marker Cd34 (relative area, expressed as % *vs.* total area: 4.6 ± 0.6 in EC^{KO} *vs.* 0.8 ± 0.3 in *WT*; *n*=4; P<0.01).

Note S9. While the vast majority of the studies with EC^{OE} mice were performed with males, some important findings were confirmed in female mice. A similar decrease in the number of medium-sized vessels was observed in EC^{OE} females compared to their *WT* littermates 4 weeks post-tamoxifen (number/area in mm^2: 5,3 ± 0,2 in EC^{OE} *vs.* 6,6 ± 0,4 in *WT*; *n*=5-6; P<0.05).

Note S10. In order to deliver additional evidence that spontaneous activation of HSCs and fibrosis upon endothelial *Zeb2* deletion is unlikely, we also looked at the longer-term effects of the deletion where mice were analysed by histology 26 weeks after tamoxifen-induced deletion. HSC activation was not induced upon long-term endothelial *Zeb2* deletion as evident from the absence of the HSC activation marker αSMA and there were no signs of fibrosis on Sirius red-stained liver cross-sections.

E. SUPPLEMENTARY REFERENCES

1. Motoike T, Loughna S, Perens E, Roman BL, Liao W, Chau TC, Richardson CD, Kawate T, Kuno J, Weinstein BM, Stainier DY, Sato TN. Universal GFP reporter for the study of vascular development. *Genesis* 2000;**28**:75-81.
2. Nishizaki Y, Takagi T, Matsui F, Higashi Y. SIP1 expression patterns in brain investigated by generating a SIP1-EGFP reporter knock-in mouse. *Genesis* 2014;**52**:56-67.
3. Wang Y, Nakayama M, Pitulescu ME, Schmidt TS, Bochenek ML, Sakakibara A, Adams S, Davy A, Deutsch U, Luthi U, Barberis A, Benjamin LE, Makinen T, Nobes CD, Adams RH. Ephrin-B2 controls VEGF-induced angiogenesis and lymphangiogenesis. *Nature* 2010;**465**:483-486.
4. Higashi Y, Maruhashi M, Nelles L, Van de Putte T, Verschueren K, Miyoshi T, Yoshimoto A, Kondoh H, Huylebroeck D. Generation of the floxed allele of the SIP1 (Smad-interacting protein 1) gene for Cre-mediated conditional knockout in the mouse. *Genesis* 2002;**32**:82-84.
5. Tatari MN, De Craene B, Soen B, Taminau J, Vermassen P, Goossens S, Haigh K, Cazzola S, Lambert J, Huylebroeck D, Haigh JJ, Berx G. ZEB2-transgene expression in the epidermis compromises the integrity of the epidermal barrier through the repression of different tight junction proteins. *Cell Mol Life Sci* 2014;**71**:3599-3609.
6. Sousa VH, Miyoshi G, Hjerling-Leffler J, Karayannis T, Fishell G. Characterization of Nkx6-2-derived neocortical interneuron lineages. *Cereb Cortex* 2009;**19 Suppl 1**:i1-10.
7. Vandersmissen I, Craps S, Depypere M, Coppiello G, van Gastel N, Maes F, Carmeliet G, Schrooten J, Jones EA, Umans L, Devlieger R, Koole M, Gheysens O, Zwijsen A, Aranguren XL, Luttun A. Endothelial Msx1 transduces hemodynamic changes into an arteriogenic remodeling response. *J Cell Biol* 2015;**210**:1239-1256.
8. Coppiello G, Collantes M, Sirerol-Piquer MS, Vandenwijngaert S, Schoors S, Swinnen M, Vandersmissen I, Herijgers P, Topal B, van Loon J, Goffin J, Prosper F, Carmeliet P, Garcia-Verdugo JM, Janssens S, Penuelas I, Aranguren XL, Luttun A. Meox2/Tcf15 heterodimers program the heart capillary endothelium for cardiac fatty acid uptake. *Circulation* 2015;**131**:815-826.
9. de Haan W, Oie C, Benkheil M, Dheedene W, Vinckier S, Coppiello G, Aranguren XL, Beerens M, Jaekers J, Topal B, Verfaillie C, Smedsrod B, Luttun A. Unraveling the transcriptional determinants of liver sinusoidal endothelial cell specialization. *Am J Physiol Gastrointest Liver Physiol* 2020;**318**:G803-G815.
10. Picelli S, Bjorklund AK, Faridani OR, Sagasser S, Winberg G, Sandberg R. Smart-seq2 for sensitive full-length transcriptome profiling in single cells. *Nat Methods* 2013;**10**:1096-1098.
11. Bolger AM, Lohse M, Usadel B. Trimmomatic: a flexible trimmer for Illumina sequence data. *Bioinformatics* 2014;**30**:2114-2120.
12. Kim D, Langmead B, Salzberg SL. HISAT: a fast spliced aligner with low memory requirements. *Nat Methods* 2015;**12**:357-360.

13. Anders S, Pyl PT, Huber W. HTSeq--a Python framework to work with high-throughput sequencing data. *Bioinformatics* 2015;**31**:166-169.

14. Risso D, Ngai J, Speed TP, Dudoit S. Normalization of RNA-seq data using factor analysis of control genes or samples. *Nat Biotechnol* 2014;**32**:896-902.

15. Risso D, Schwartz K, Sherlock G, Dudoit S. GC-content normalization for RNA-Seq data. *BMC Bioinformatics* 2011;**12**:480.

16. Robinson MD, McCarthy DJ, Smyth GK. edgeR: a Bioconductor package for differential expression analysis of digital gene expression data. *Bioinformatics* 2010;**26**:139-140.

17. Chen EY, Tan CM, Kou Y, Duan Q, Wang Z, Meirelles GV, Clark NR, Ma'ayan A. Enrichr: interactive and collaborative HTML5 gene list enrichment analysis tool. *BMC Bioinformatics* 2013;**14**:128.

18. Browaeys R, Saelens W, Saeys Y. NicheNet: modeling intercellular communication by linking ligands to target genes. *Nat Methods* 2020;**17**(2):159-162.

19. Beerens M, Aranguren XL, Hendrickx B, Dheedene W, Dresselaers T, Himmelreich U, Verfaillie C, Luttun A. Multipotent Adult Progenitor Cells Support Lymphatic Regeneration at Multiple Anatomical Levels during Wound Healing and Lymphedema. *Sci Rep* 2018;**8**:3852.

20. Xie G, Wang X, Wang L, Wang L, Atkinson RD, Kanel GC, Gaarde WA, Deleve LD. Role of differentiation of liver sinusoidal endothelial cells in progression and regression of hepatic fibrosis in rats. *Gastroenterology* 2012;**142**:918-927 e916.

21. Demene C, Deffieux T, Pernot M, Osmanski BF, Biran V, Gennisson JL, Sieu LA, Bergel A, Franqui S, Correas JM, Cohen I, Baud O, Tanter M. Spatiotemporal Clutter Filtering of Ultrafast Ultrasound Data Highly Increases Doppler and fUltrasound Sensitivity. *IEEE Trans Med Imaging* 2015;**34**:2271-2285.

22. Bonnefous O, Pesque P. Time domain formulation of pulse-Doppler ultrasound and blood velocity estimation by cross correlation. *Ultrason Imaging* 1986;**8**:73-85.

23. Walter TJ, Cast AE, Huppert KA, Huppert SS. Epithelial VEGF signaling is required in the mouse liver for proper sinusoid endothelial cell identity and hepatocyte zonation in vivo. *Am J Physiol Gastrointest Liver Physiol* 2014;**306**:G849-862.

24. Halpern KB, Shenhav R, Massalha H, Toth B, Egozi A, Massasa EE, Medgalia C, David E, Giladi A, Moor AE, Porat Z, Amit I, Itzkovitz S. Paired-cell sequencing enables spatial gene expression mapping of liver endothelial cells. *Nat Biotechnol* 2018;**36**:962-970.

25. Cheng X, Kim SY, Okamoto H, Xin Y, Yancopoulos GD, Murphy AJ, Gromada J. Glucagon contributes to liver zonation. *Proc Natl Acad Sci U S A* 2018;**115**:E4111-E4119.

26. Winkler M, Staniczek T, Kurschner SW, Schmid CD, Schonhaber H, Cordero J, Kessler L, Mathes A, Sticht C, Nessling M, Uvarovskii A, Anders S, Zhang XJ, von Figura G, Hartmann D, Mogler C, Dobreva G, Schledzewski K, Geraud C, Koch PS, Goerdt S. Endothelial GATA4 controls liver fibrosis and regeneration by preventing a pathogenic switch in angiocrine signaling. *J Hepatol* 2021;**74**(2):380-393.
